# Non-invasive lung cancer diagnosis and prognosis based on multi-analyte liquid biopsy

**DOI:** 10.1186/s12943-021-01323-9

**Published:** 2021-01-29

**Authors:** Kezhong Chen, Jianlong Sun, Heng Zhao, Ruijingfang Jiang, Jianchao Zheng, Zhilong Li, Jiaxi Peng, Haifeng Shen, Kai Zhang, Jin Zhao, Shida Zhu, Yuying Wang, Fan Yang, Jun Wang

**Affiliations:** 1 Department of Thoracic Surgery, Peking University People’s Hospital, Beijing, 100044 China; 2 Envelope Health Biotechnology Co. Ltd., BGI-Shenzhen, Shenzhen, 518083 China; 3 BGI Genomics, BGI-Shenzhen, Shenzhen, 518083 China; 4 Shenzhen Engineering Laboratory for Innovative Molecular Diagnostics, BGI-Shenzhen, Shenzhen, 518120 China

**Keywords:** Lung cancer, Diagnosis, Prognosis, Liquid biopsy, cfDNA, Mutation, DNA methylation, Multi-analyte

## Main text

Lung cancer (LC) is the leading cause of death in many countries including China. The stage at which LC is diagnosed has a significant impact on prognosis. However, timely detection of LC remains difficult since patients are often asymptomatic at early stages. Low-dose computed tomography (LDCT) is the most extensively recommended LC screening method currently, but it poses radiation risks and only a small fraction of the nodules detected are true lung cancers. In clinical practice, it remains a challenge to differentiate malignant tumors from benign solitary pulmonary nodules, which may greatly benefit from non-invasive diagnostic tools. TNM stage currently remains the most widely used prognostic tool in lung cancer. However, the variability of survival within staging groups suggests that search for additional prognostic parameters is necessary. Molecular alterations such as cancer driver gene mutational status and expression signatures have been implicated in LC prognosis; meanwhile, there have been emerging evidence that support the prognostic value of epigenetic alterations, which remains to be fully elucidated.

Circulating tumor DNA (ctDNA) in plasma of cancer patients provides valuable information for cancer genome and also holds great promise for non-invasive cancer detection [1, 2]. However, since ctDNA is diluted by abundant circulating cell-free DNA (cfDNA) of noncancerous origins, its detection poses significant challenges especially during early stages of cancer when the tumor mass is small. In this study, we developed a set of experimental and computational tools to measure both genetic and epigenetic signals from plasma cfDNA of LC patients as well as patients bearing benign lung nodules (BLN) using high-throughput sequencing [3], aiming to explore the potential utility of blood-based biomarkers for LC diagnosis and prognosis.

## Results and discussions

### Targeted ultra-deep sequencing detected distinct mutational spectra of plasma cfDNA and WBC gDNA

**Fig. 1:**
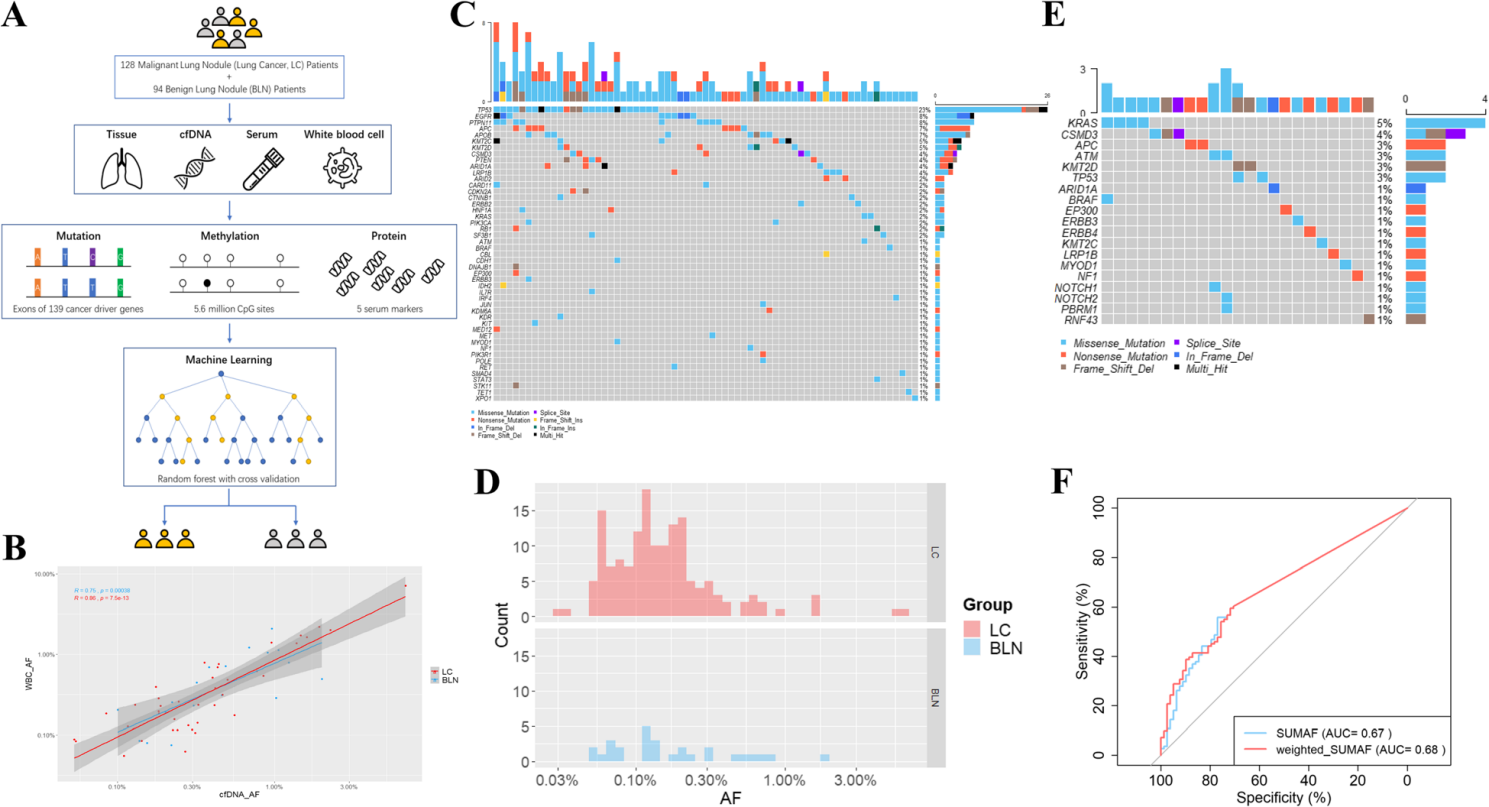
Study design and classification models based on variants detected in plasma cfDNA after filtering with matched WBC sample for shared variants. **a** Schematic view of the study design. **b** Pearson correlation of AF in cfDNA (x-axis, log scale) and AF in matched WBC gDNA (y-axis, log scale). Each point represents one variant detected in matched cfDNA and WBC gDNA samples from the same patient. **c** Oncoplot showing the 153 mutations detected in 67 out of 111 (60.36%) LC samples. Fourty-five LC samples without any mutation detected were not shown. Each column represents a sample and each row a different gene. The upper barplot represents the frequency of mutations for each sample, and the right barplot represents the frequency of mutations for each gene. Samples are ordered by the most mutated genes. **d** Allele fractions (x-axis, log scale) of mutations detected in plasma cfDNA of BLN patients (blue) and LC patients (red). **e** Oncoplot of the 28 mutations detected in 23 out of 78 (29.49%) BLN samples. 55 BLN samples without any mutation detected were not shown. **f** Predictive models to distinguish LC from BLN based on mutations detected. SUMAF (blue): the sum of AFs. Weighted_SUMAF (red): the weighted sum of AFs. The AUC of SUMAF model is 0.67 with 55.9% sensitivity and 76.9% specificity. The AUC of weighted_SUMAF model is 0.68 with 59.5% sensitivity and 71.8% specificity

A cohort of 128 LC patients represented a natural tumor stage distribution (66% of the cases were stage 0 or stage I) and 94 BLN patients were enrolled in this study (Fig. 1a and Table 1). To detect genomic sequence alterations, we performed targeted ultra-deep next-generation sequencing (NGS) on plasma cfDNA extracted from 111 LC patients and 78 BLN patients using a panel covering exons of 139 cancer driver genes selected based on TCGA and COSMIC databases (Supplementary Table 1 and 2, and Supplementary Fig. 1). Adaptors that contained 6 bp duplex unique molecule identifiers (UMI) were used in the library preparation procedure to enable subsequent removal of PCR duplicates and error-correction based on consensus generation. A set of stringent thresholds were then applied to identify the most reliable somatic variants (See Methods for details). In total, 193 and 46 mutations were detected in 75 (68%) LC patients and 33 (42%) BLN plasma cfDNA, respectively (Supplementary Fig. 2 and 3).

**Table 1:** Clinicopathological characteristics of the patients enrolled in this study

		LC (***N*** = 128)		BLN (***N*** = 94)		***p***-value
		Number	Percentage	Number	Percentage	
**Gender**	**Female**	**53**	**41%**	**48**	**51%**	**0.15** **(chi-squared test)**
	**Male**	**75**	**59%**	**46**	**49%**	
**Age**	**Median ± SD (Range)**	**63.00 ± 11.58 (30–86)**		**55.00 ± 10.49 (18–79)**		**1.00E-05** **(Student’s t-test)**
**Nodule Size (cm)**	**Median ± SD (Range)**	**2.00 ± 1.35 (0.20–6.50)**		**1.25 ± 1.14 (0.35–5.75)**		**1.22E-03** **(Student’s t-test)**
**Histology**	**LUAD**	**97**	**76%**			
	**LUSC**	**23**	**18%**			
	**LCC**	**3**	**2%**			
	**SCLC**	**5**	**4%**			
	**Inflammatory Lesion**			**31**	**33%**	
	**Granulomatous Inflammation**			**12**	**13%**	
	**Atypical Adenomatous Hyperplasia**			**10**	**11%**	
	**Atypical Hyperplasia**			**10**	**11%**	
	**Others**			**31**	**33%**	
**Stage**	**0**	**2**	**2%**			
	**IA**	**54**	**42%**			
	**IB**	**29**	**23%**			
	**II**	**17**	**13%**			
	**III**	**19**	**15%**			
	**IV**	**7**	**5%**			
**Smoking History**	**Current-Smoker**	**29**	**23%**	**15**	**16%**	**0.02** **(chi-squared test)**
	**Ex-Smoker**	**21**	**16%**	**8**	**9%**	
	**Non-smokers**	**77**	**60%**	**70**	**74%**	
	**Unknown**	**1**	**1%**	**1**	**1%**	
**Smoking Levels (pack-years)**	**Median ± SD (Range)**	**37.50 ± 27.96 (2–120)**		**20.00 ± 15.02 (5–60)**		**0.01** **(Student’s t-test)**

Smoking Levels: Among Ever-smokers only. *LUAD* lung adenocarcinoma, *LUSC* lung squamous cell carcinoma, *LCC* large cell carcinoma, *SCLC* small cell lung carcinoma

Since some variants might derive from clonal hematopoiesis (CH) and confound the mutational analysis [4], genomic DNA (gDNA) of white blood cell (WBC) from cfDNA mutation-positive participants was also sequenced. Non-synonymous variants were detected in WBC of 73 (97%) LC patients and 33 (100%) BLN patients respectively (Supplementary Fig. 4 and 5). Among WBC-shared cfDNA variants, the most frequently mutated genes included *TP53*, *CBL*, *APOB*, and *CSMD3* for LC plasma, and *CBL*, *CSMD3*, and *STAT3* for BLN plasma (Supplementary Fig. 6). Moreover, allele frequencies (AFs) of variants shared by cfDNA and matched WBC samples were highly correlated (Fig. 1b), suggesting that these mutations indeed originated from WBC and should be removed for downstream analysis [5]. The percentages of cfDNA variants matching corresponding WBC sample were 20.7% (40 out of 193) for LC cfDNA and 39.1% (18 out of 46) for BLN cfDNA, suggesting that a significant portion of cfDNA variants was derived from CH, especially in BLN plasma (*p* = 8.89E-03, chi-squared test). Notably, a number of these mutations were hotspot mutations of cancer driver genes (Supplementary Fig. 7), suggesting that CH variants may significantly confound cfDNA analysis if not analyzed in parallel.

After filtering for variants potentially derived from CH, 153 variants remained in 67 (out of 111, 60.36%) LC cfDNA samples (Fig. 1c and Supplementary Table 3), with AFs ranging from 0.03 to 6.00% (median was 0.13%, Fig. 1d and Supplementary Fig. 8). *TP53* was the most commonly mutated gene in LC plasma (mutated in 23% of LC cfDNA samples) followed by *EGFR* (8%), *PTPN11* (8%), *APC* (7%), *APOB* (7%), *KMT2C* (5%)*,* and *KMT2D* (5%) (Fig. 1c and Supplementary Fig. 9 and 10). Smoking is an important risk factor for lung cancer. We observed that within LC patients, smokers appeared to carry a higher mutation burden in plasma cfDNA than never-smokers (Supplementary Fig. 11). 28 mutations remained in 23 (out of 78, 29.49%) BLN cfDNA samples (Fig. 1e and Supplementary Table 4), although the fraction of positive samples was much less, compared to LC plasma (29.49% vs. 60.36%, *p* = 2.87E-05, chi-squared test). These mutations had AFs ranging from 0.05 to 1.91% (Fig. 1d). The most frequently mutated genes in BLN plasma were *KRAS* (5%), *CSMD3* (4%), *APC* (3%), *ATM* (3%), *KMT2D* (3%), and *TP53* (3%) (Fig. 1e), representing a distinct mutational spectrum from LC cfDNA. Notably, 39.3% (11 out of 28) of these were COSMIC hotspot mutations (Supplementary Table 4). These results revealed that, in contrast to common belief, plasma cfDNA from BLN patients also carried genomic sequence alterations including mutations in cancer driver genes, albeit less frequently. These alterations could have arisen from somatic clonal expansions in normal tissues [6]. Also, it was noted that some of the benign lesions included in our study were regarded as premalignant lung lesions, such as atypical adenomatous hyperplasia (AAH, 11% of BLN cases in our study). Previous study showed that AAH indeed harbored cancer driver mutations, such as those in gene *KRAS*, *BRAF*, *APC*, *KMT2D*, and *TP53*, and these mutations could be readily detected in matched plasma cfDNA [7]. Taken together, these results highlight the potential challenges for differentiating malignant versus benign plasma based on the cfDNA mutation spectrum.

### Classification models based on somatic mutations to distinguish LC from BLN

Next, we investigated whether LC plasma cfDNA had a stronger mutational burden than that of BLN patients. To quantify the cfDNA mutational burden, we constructed a mutation score for each cfDNA sample as either a simple summation of the allele fractions of all variants identified (SUMAF), or a weighted sum of the allele fractions, weighting more on TCGA hotspot cancer driver mutations and COSMIC hotspot mutations (weighted SUMAF, or wSUMAF; see Methods for details). Both scoring methods produced modest classification accuracy for distinguishing LC from BLN plasma: the wSUMAF model generated an area under curve (AUC) value of 0.68 with a sensitivity of 59.5% and a specificity of 71.8% (Fig. 1f and Supplementary Fig. 12) and the SUMAF model had a similar performance. These results showed that the classification models built on mutation score alone had limited classification capability for differentiating LC and BLN plasma, contradicting some earlier studies which suggested that mutational status could be used to diagnose LC from BLN with high specificity and modest sensitivity [8], a conclusion that may have suffered from potential bias caused by limited sample sizes used. Our work obtained from a larger sample size (128 LC and 94 BLN plasma) suggested that genomic sequence alterations in cancer driver genes carried by BLN cfDNA might be more prevalent than previously thought, therefore limiting the utility of mutation-based diagnostic assays. A multi-analyte approach is more likely to improve the detection of cancer signal.

### Classification of LC and BLN plasma based on cfDNA methylation data

**Fig. 2:**
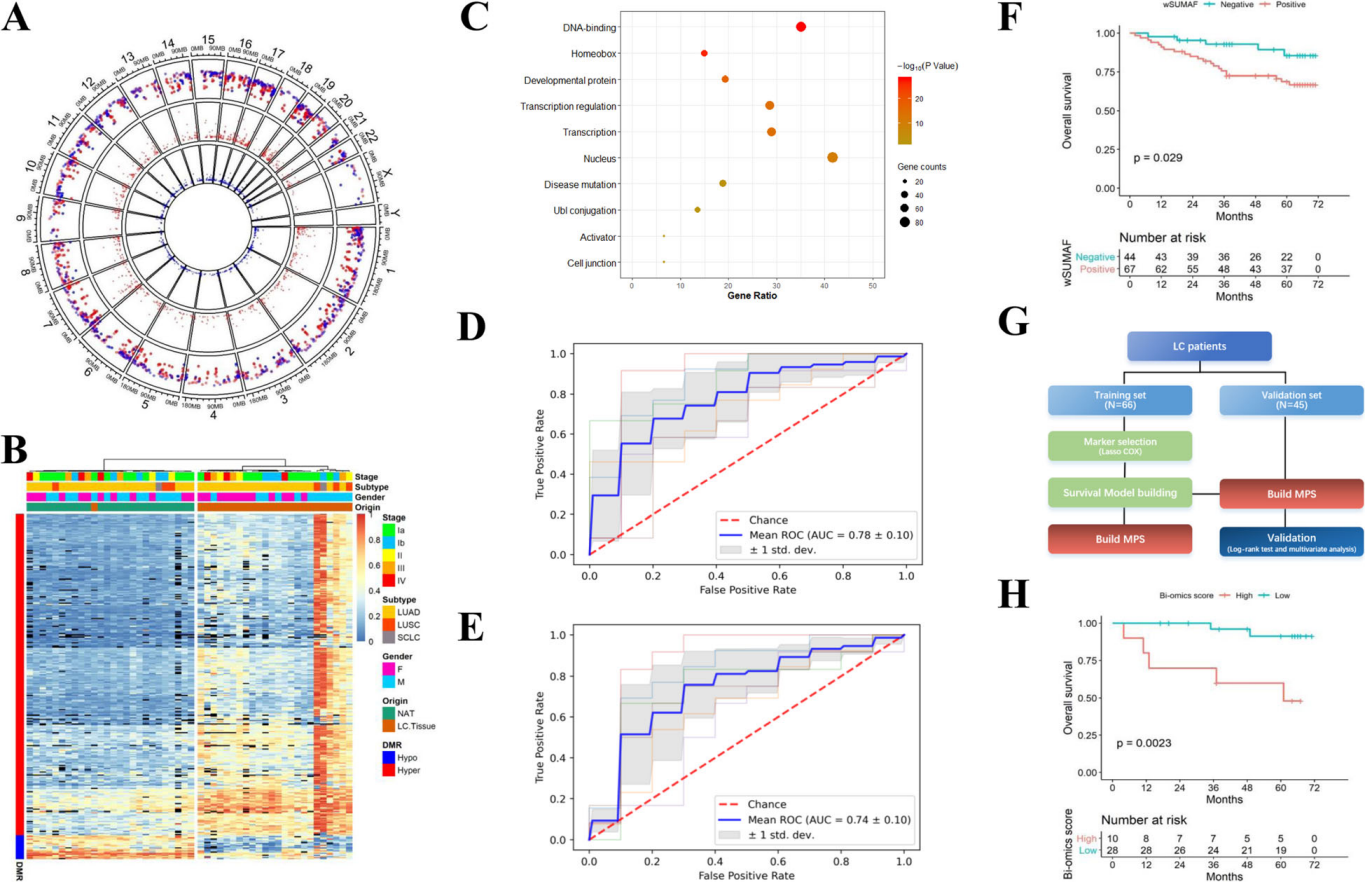
DNA methylation and multi-omics analysis. **a** Differentially methylated regions (DMRs) discovered by WGBS of LC tumor tissue and normal tissue adjacent to the tumour (NAT). Red points: Hypermethylated DMRs in LC tissues. Blue points: hypomethylated DMRs in LC tissues. From outer to inner circle: the first circle is the overview of DMRs, the second circle is the area statistics of hypermethylation regions (methy.diff> 0.2), and the third circle is the area statistics of hypomethylation regions (methy.diff<− 0.2). **b** Heatmap of the DMRs with hierarchical clustering. Block color represents the methylation β value and black represent N.A. values due to insufficient sequencing depth. **c** Functional annotation of the genes associated with the 293 hypermethylated DMRs by gene ontology (GO) terms using DAVID. **d** Predictive models based on combined mutation score, selected DMR, and serum CEA levels in tri-omics profiled samples. **e** Predictive models based on mutation score and selected DMR to distinguish LC from BLN plasma. **f** Kaplan-Meier plot on mutation-based LC prognostic model in relation to OS in the whole dataset. **g** Flow chart of methylation-based survival analyses on LC patients. **h** Kaplan-Meier plot on multi-omics-based prognostic model in relation to OS in the testing set

To identify LC-specific epigenetic changes, we performed whole-genome bisulfite sequencing (WGBS) on 25 pairs of LC tissue and normal tissue adjacent to the tumor (NAT) (Fig. 2a). Three hundred fifteen differentially methylated regions (DMRs) were identified, including 293 hype-methylated DMRs and 22 hypo-methylated DMRs (Fig. 2b; see Methods for details). There were a lot more hyper DMRs than hypo DMRs, consistent with the belief that genomic regulatory regions such as promoters of potential tumor suppressor genes undergo remarkable hypermethylation in tumorigenesis [9]. Gene ontology (GO) annotations revealed that the 293 hyper DMRs were significantly enriched for genes encoding DNA-binding domains and homeobox domains, as well as genes involved in the developmental and transcriptional regulation process (Fig. 2c). These genes are likely to be potential tumor suppressor genes, and many of which haven’t been implicated as such previously (such as *SEC31B*, *ZNF274*, and *NXPH1*). Unsupervised hierarchical clustering using the regional methylation ratio of the identified DMRs perfectly separated LC tissues and NAT with the exception of a single LC sample, highlighting the pronounced epigenetic dysregulation of LC cells (Fig. 2b).

We next performed a comprehensive analysis of 5-mC methylation profile of cfDNA for 111 LC patients and 87 BLN patients using targeted bisulfite sequencing, covering 5.6 million CpG sites (Supplementary Table 1). Compared to BLN plasma, increased methylation levels were observed in LC plasma cfDNA for hypermethylated DMRs identified from tissue sequencing, as expected (Supplementary Fig. 13). The DMR methylation levels of cfDNA appeared to be lower in smokers than never-smokers, in both the LC and BLN group (Supplementary Fig. 14). To test the diagonostic value of methylation markers, we first built a random forest model with 6-fold cross-validation (CV) that classified LC from BLN plasma based on hyper DMRs, which achieved an AUC of 0.71 (Supplementary Fig. 15). A feature selection process was then carried out to minimize the number of DMR markers while maintaining the performance. By selecting features with the highest feature importance in each fold of the CV, a model consisting of 47 DMRs was obtained, achieving a similar performance with an AUC of 0.71 while reducing the feature size by 84.0%, and still outperforming models based on mutation status alone (Supplementary Fig. 16). These results indicated that LC-specific methylation changes carried by plasma cfDNA could be effective biomarkers for diagnosing lung cancers versus benign lesions. Here, the difference in model performance comparing to previous study on methylation markers could be attributed to the different study populations and cfDNA analysis methods. The smaller cohort size in the previous study may have also caused over-fitting and/or over-estimation of the model performance.

### Multi-omics analysis to differentiate LC from BLN plasma

Next, we attempted to integrate multi-omics features of the cfDNA to further improve the diagnostic power of our classification model among samples with complete measurements (Supplementary Table 1). Indeed, among 91 LC and 71 BLN cfDNA samples that underwent both genetic and epigenetic profiling, we found that models that combined 59 most informative DMRs and the wSUMAF mutation score achieved a CV-AUC of 0.77 with a sensitivity of 76.1% and a specificity of 59.2%, compared to an AUC of 0.68 achieved by mutation score alone (DeLong *p*-value< 0.01) and an AUC of 0.74 achieved by methylation features alone (DeLong p-value = 0.85) (Supplementary Fig. 17).

We then tried to incorporate serum protein markers into the classification model. Among 5 serum markers measured, including CEA, CYFRA21-1, NSE, CA19–9, and CA125, only CEA level appeared to be significantly higher in LC patients than BLN patients (*p* = 0.04, Student’s t-test), producing a modest classification AUC of 0.66 (Supplementary Fig. 18 and 19). The multi-omics predictive models based on the combination of wSUMAF mutation score, regional methylation ratio of 54 selected DMRs, and the serum CEA level achieved an AUC of 0.78, with 76.9% sensitivity and 58.3% specificity (Fig. 2d) on the set of samples with complete measurements of all three types of analytes (74 LC and 60 BLN). This result showed a further improvement in diagnostic accuracy compared to the models without CEA in the same set of samples (AUC = 0.74, DeLong *p*-value = 0.02) (Fig. 2e). To our knowledge, this is the first proof-of-concept study to demonstrate that genetic, epigenetic, and proteomic analytes could be combined to improve the performance of liquid biopsy-based diagnostic assay for LC. Here, the mediocre performance of the final multi-omics model could be attributed to the fact that a large proportion of the LC cases (*n* = 85, 66%) included in the study were stage 0 or stage I and the majority of the cases (*n* = 97, 75%) were lung adenocarcinoma (LUAD) which were previously suggested to release less ctDNA into the bloodstream compared to lung squamous cell carcinoma (LUSC) (*n* = 23, 18% in this study). A recent study which used methylation-based ctDNA markers for non-invasive detection of multiple cancer types also found low sensitivies for early-stage lung cancers [9]; another recent study which integrated multiple genomic features to develop a ctDNA-based assay for LC detection also reported modest performance for stage I and II lung cancers (the Lung-CLiP model; AUC = 0.69–0.71) [5], corroborating our findings. Care needs to be taken when apply the findings presented in this study to cohort with different clinicopathological characteristics, and additional study with larger sample size would be necessary to validate current findings.

### cfDNA mutation burden and methylation status as prognostic factors for LC

We first tested whether mutational status (wSUMAF, < 0 vs. > 0) was associated with LC overall survival (OS). We found that a higher mutation burden was associated with a significantly worse OS (Fig. 2f). This association was also significant among stage I patients (Supplementary Fig. 20). Next, we attempted to identify potential methylation-based prognostic biomarkers (Fig. 2g) and to incorporate these markers into the prediction model for prognostic stratification of the LC patients. Previously, multiple methylation-based prognostic classifiers had been reported for lung cancer, however, the reported markers were mostly inconsistent. The inconsistency could be explained by limited sample sizes, variations in study design, as well as different detection methods used. We first obtained corresponding coefficients for candidate features using penalized Cox regression among training set and incorporated these features into the model (Supplementary Fig. 21). The methylation-based prognostic score (MPS) was then calculated for each individual as a weighted sum of the methylation level of 12 selected DMRs (Supplementary Table 5) multipliedby their corresponding coefficients. The MPS was then combined with the mutation score as the bi-omics prognosis score. Patients with a high mutational burden and a high MPS were categorized as the high prognosis score group, which had a significantly worse OS than the low prognosis score group in the testing set (Fig. 2h). Finally, to avoid information loss due to categorization, we modeled both mutation score and MPS continuously in two multivariate Cox proportional hazard models on wSUMAF only, as well as in combination with MPS. We found the latter model achieved a higher AUC (Likelihood ratio test *p*-value = 0.27, Supplementary Fig. 22), although the difference between the two models was not statistically significant, which may be attributed to the limited number of samples included in the tesing set (*n* = 38). Taken together, these results suggest that integrated genomic features have the potential to be used as better prognostic biomarkers for LC [10].

## Conclusion

In summary, we performed comprehensive genetic and epigenetic profiling of cfDNA from lung cancer patients and individuals bearing benign lung lesions. We found that the combination of genetic and epigenetic features of cfDNA along with serum protein marker CEA showed the best classification capability to differentiate the malignant vs. benign cases. Also, an integrated model that combined cfDNA mutational status and methylation-based prognostic markers has potential to improve prediction for lung cancer survival. As blood sample is relatively easily to collect for detection in distinct clinical scenarios than imagings, our results highlight the possibility of multi-analyte blood based assay for non-invasive lung cancer diagnosis and prognosis.

## Supplementary Information

**Additional file 1: Supplementary Figures.**

**Additional file 2: Supplementary Tables.**

**Additional file 3. Methods.**

## Data Availability

The data reported in this study are alsoavailable in the CNGB Nucleotide Sequence Archive (CNSA: https://db.cngb.org/cnsa; accession number CNP 0001236).

## Abbreviations

AAH: Atypical adenomatous hyperplasia
AFs: Allele frequencies
AUC: area under the curve
BLN: benign lung lesion
cfDNA: Cell-free DNA
CH: Clonal hematopoiesis
ctDNA: Circulating tumor DNA
CV: Cross-validation
DMR: Differentially methylated region
gDNA: Genomic DNA
GO: Gene ontology
LC: Lung cancer
LDCT: Low-dose computed tomography
LUAD: Lung adenocarcinoma
LUSC: Lung squamous cell carcinoma
MPS: Mmethylation-based prognostic score
NAT: Normal tissue adjacent to the tumor
OS: Overall survival
TCGA: The Cancer Genome Atlas
UMI: Unique molecule identifiers
WBC: White blood cell
WGBS: Whole-genome bisulfite sequencing
wSUMAF: Weighted SUMAF
