# Supplementary material for: Non-invasive lung cancer diagnosis and prognosis based on multi-analyte liquid biopsy
Source: Mol Cancer. 2021 Jan 29;20:23. doi: 10.1186/s12943-021-01323-9 (PMC7844900; doi:10.1186/s12943-021-01323-9)
Supplement: Supplementary file 1 — Additional file 1: Supplementary Figures. [file 12943_2021_1323_MOESM1_ESM.docx]

**Supplementary Figure 1:** **Deduped average sequencing depth on target of LC (red) and BLN (blue) patients’ plasma cfDNA.** Depth (Y-axis) was plotted against the amount of cfDNA input (X-axis, log scale).

**A**


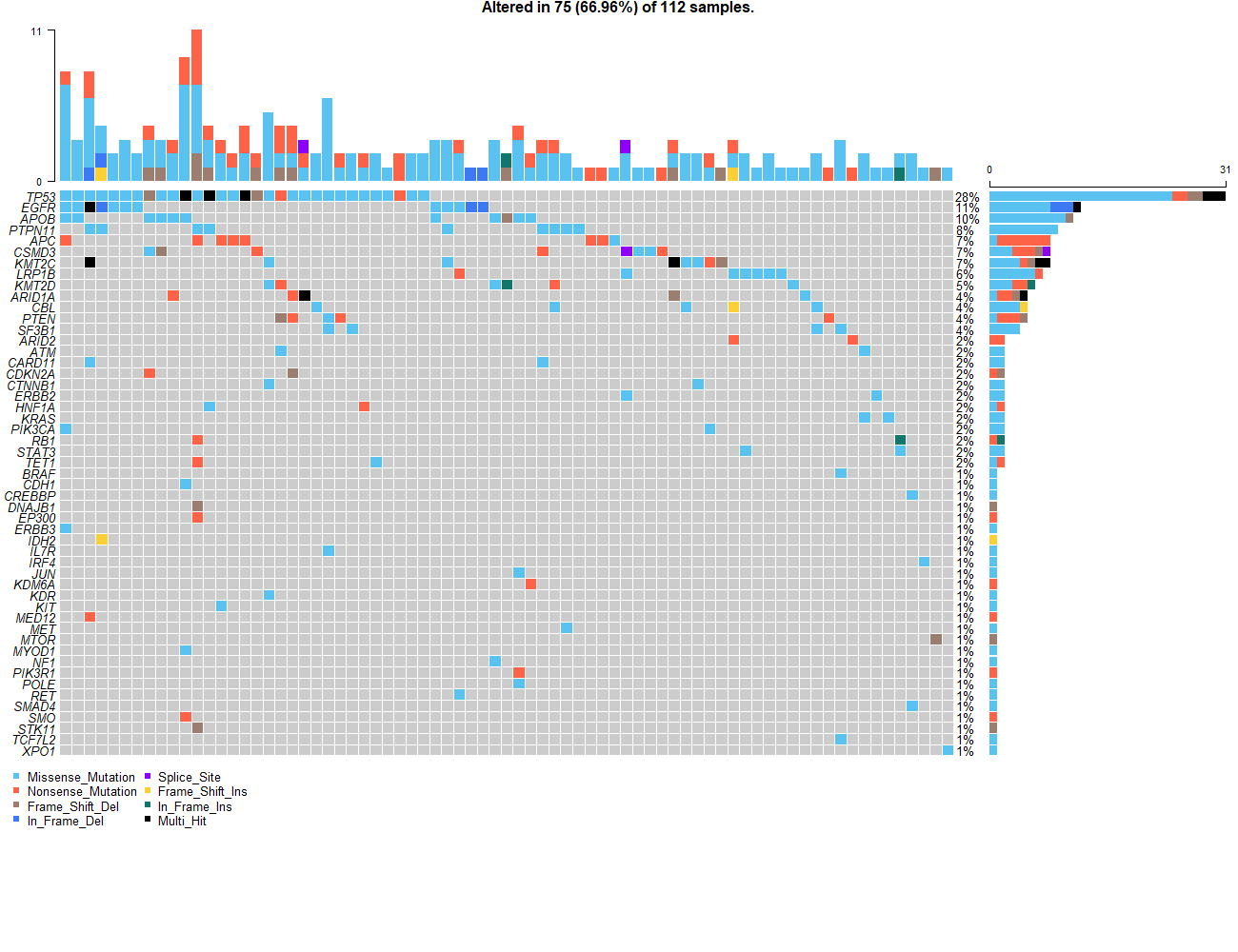


**B**

**
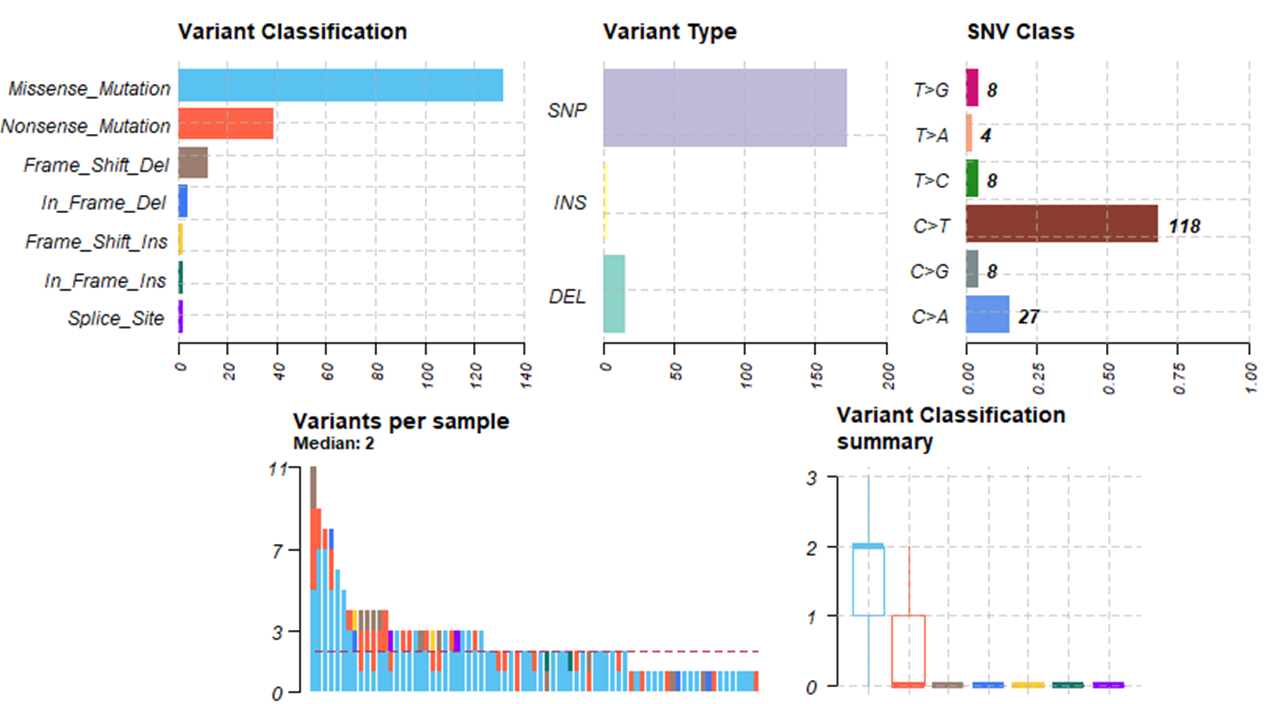
**

**Supplementary Figure 2: Somatic mutations in plasma cfDNA of LC patients before filtering for WBC shared variants.** (A) Oncoplot of the 193 mutations detected in 75 out 111 (67.57%) LC samples. 36 LC samples without any mutation detected were not drawn. Upper panel: mutation number per sample. Right panel: number and percentage of samples harbored mutations of this gene. Variant classification was represented by different colors as demonstrated at the lower left corner. (B) Summary of the 193 mutations. Upper panel from left to right: Variant classification, Variant Type, and SNV Class. Lower panel from left to right: Variants per sample and Variant classification summary.

**A**


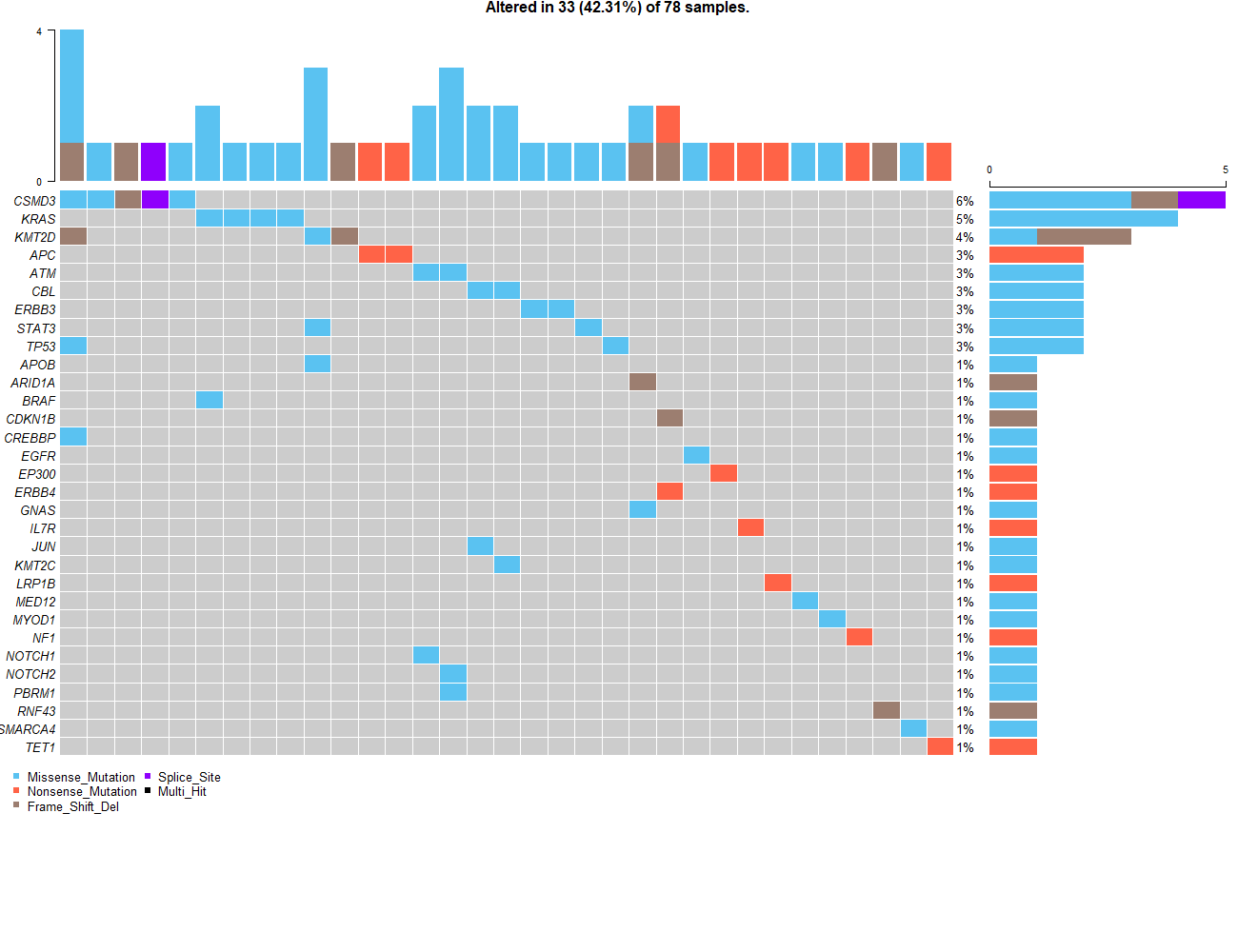


**B**

**
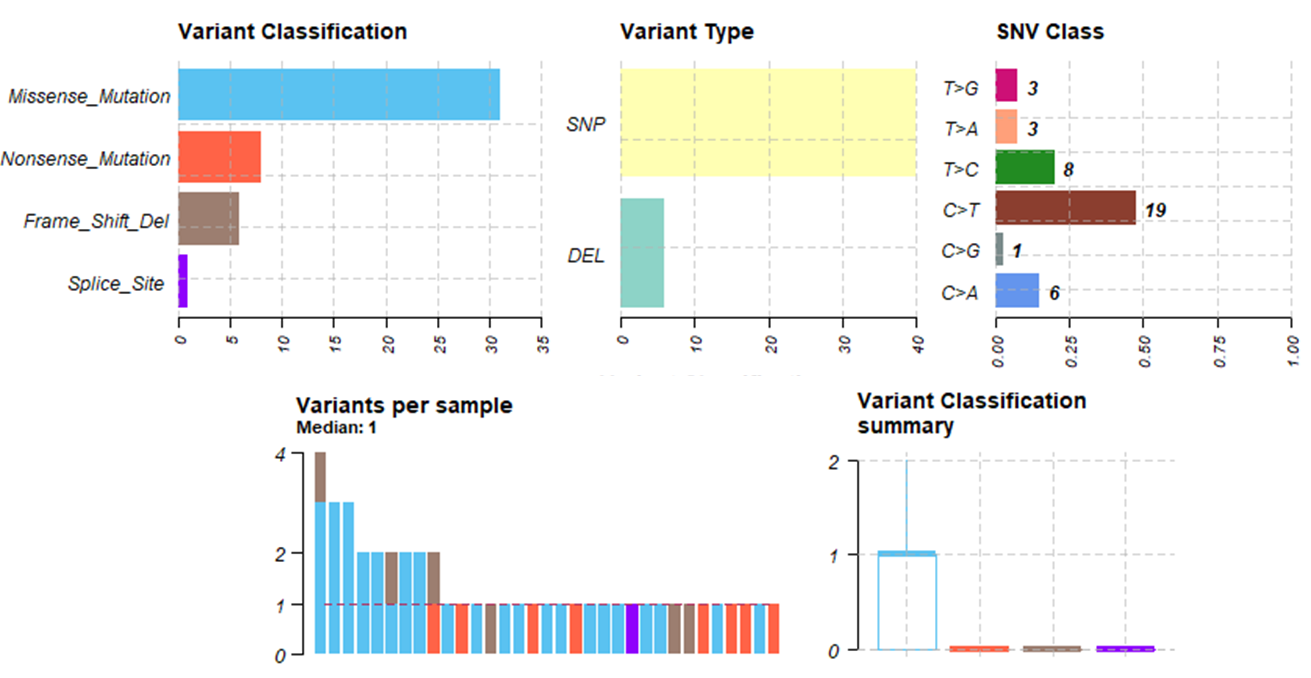
**

**Supplementary Figure 3: Somatic mutations in plasma cfDNA of BLN patients before filtering for WBC shared variants.** (A) Oncoplot of the 46 mutations detected in 33 out 78 (42.31%) BLN samples. 45 BLN samples without any mutation detected were not drawn. Upper panel: mutation number per sample. Right panel: number and percentage of samples harbored mutations of this gene. Variant classification was represented by different colors as demonstrated at the lower left corner. (B) Summary of the 46 mutations. Upper panel from left to right: Variant classification, Variant Type, and SNV Class. Lower panel from left to right: Variants per sample and Variant classification summary.

**A**


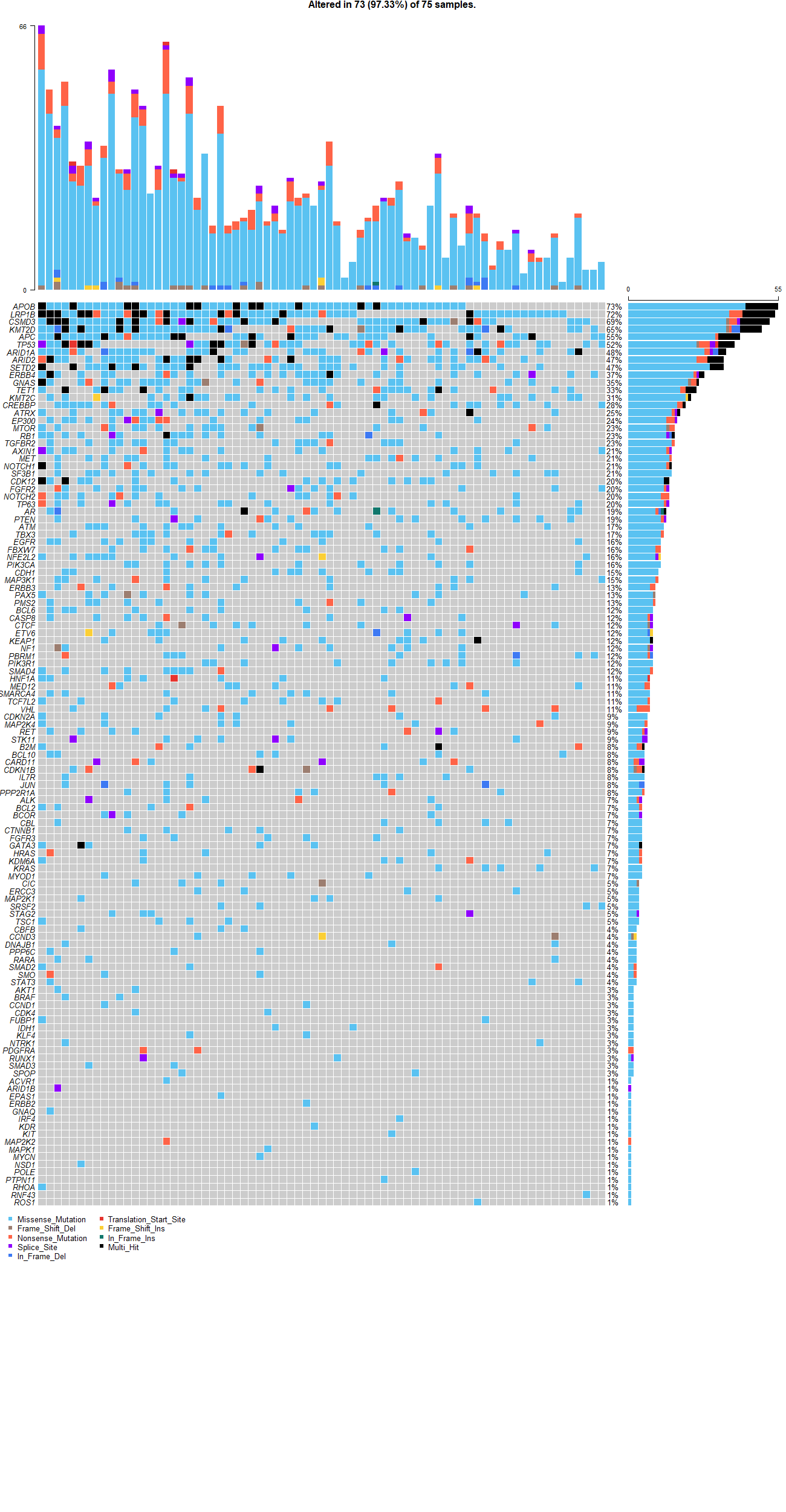


**B**

**
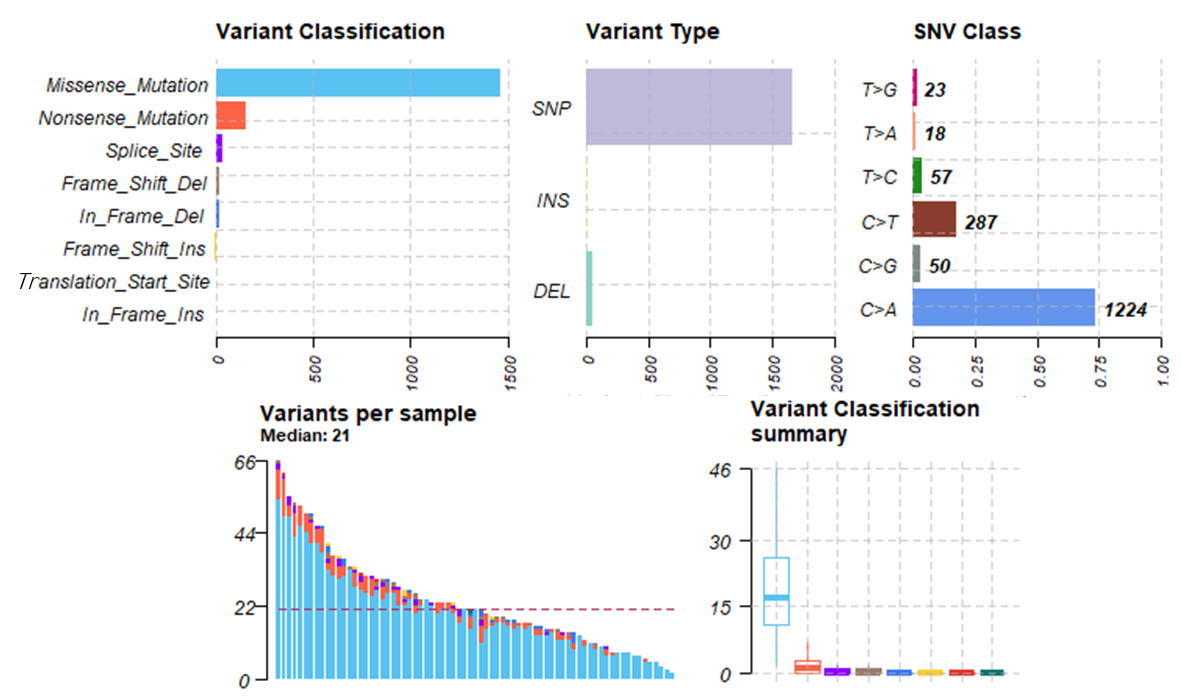
**

**Supplementary Figure 4: Non-synonymous mutations in WBC gDNA of 75 plasma cfDNA mutation-positive LC patients.** (A) Oncoplot of the 1,709 mutations detected in 73 out 75 (97.33%) LC gDNA samples. 2 LC samples without any mutation detected were not drawn. Upper panel: mutation number per sample. Right panel: number and percentage of samples harbored mutations of this gene. Variant classification was represented by different colors as demonstrated at the lower left corner. (B) Summary of the 1,709 mutations. Upper panel from left to right: Variant classification, Variant Type, and SNV Class. Lower panel from left to right: Variants per sample and Variant classification summary.

**A**


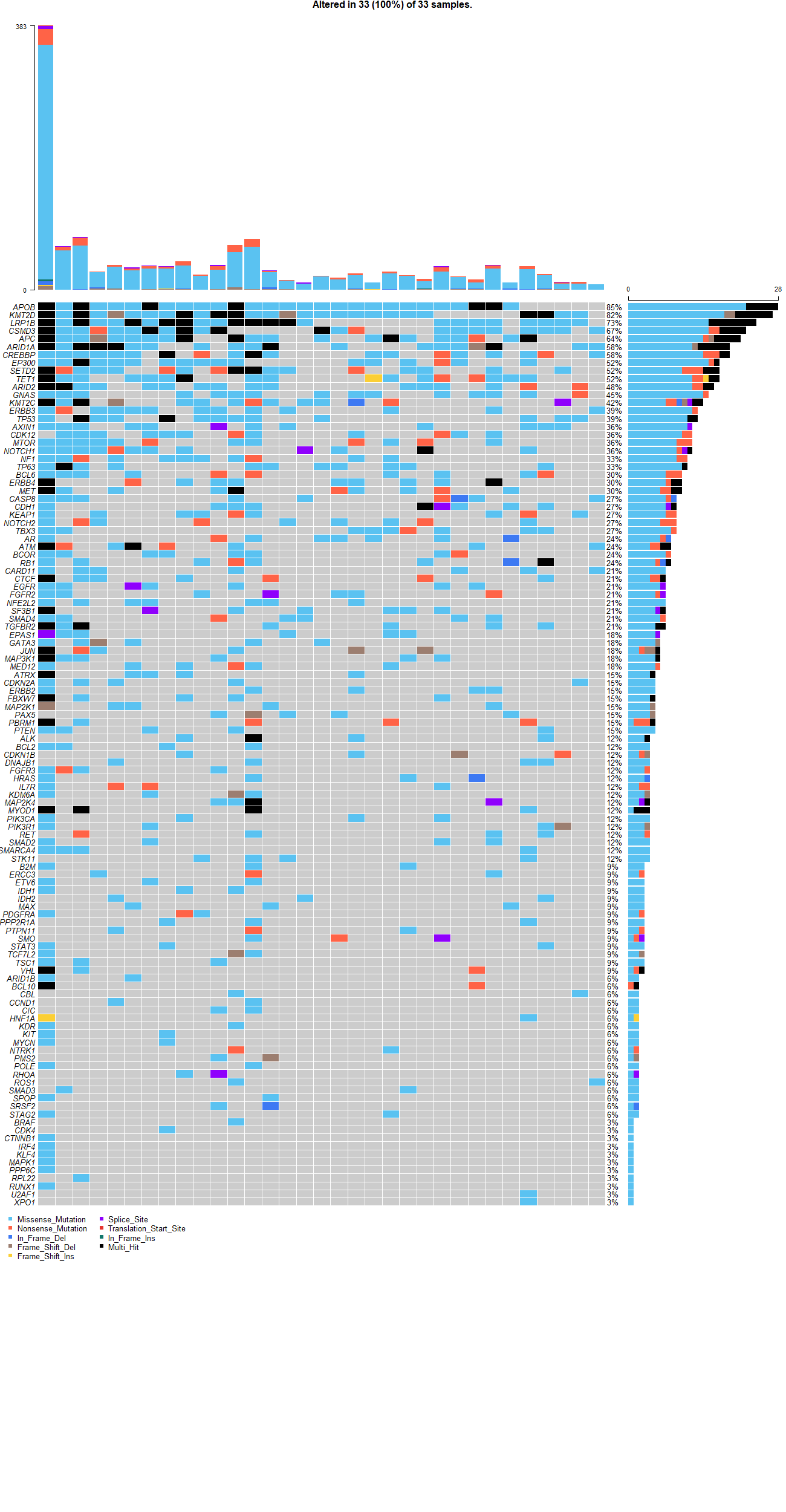


**B**

**
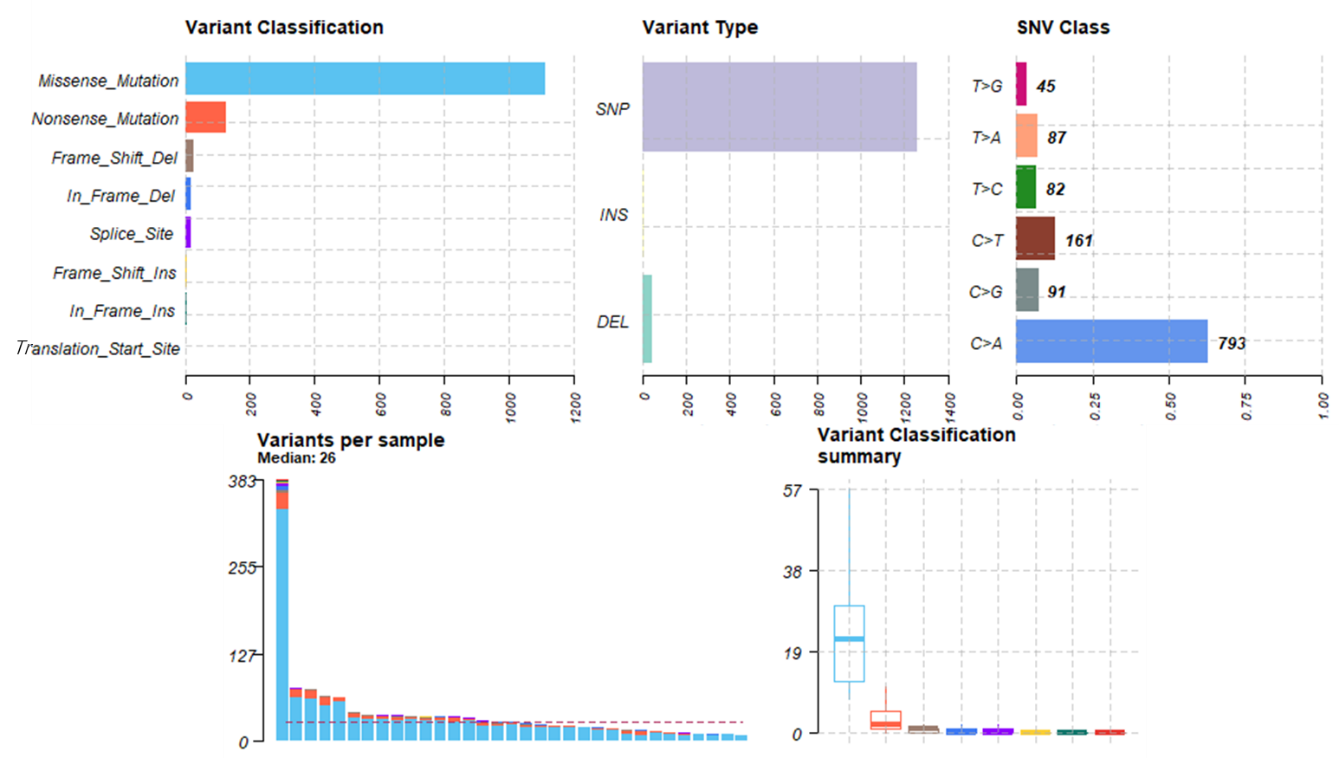
**

**Supplementary Figure 5: Non-synonymous mutations in WBC gDNA of 33 plasma cfDNA mutation-positive BLN patients.** (A) Oncoplot of the 1,311 mutations detected in 33 out 33 (100%) BLN samples. Upper panel: mutation number per sample. Right panel: number and percentage of samples harbored mutations of this gene. Variant classification was represented by different colors as demonstrated at the lower left corner. (B) Summary of the 1,311 mutations. Upper panel from left to right: Variant classification, Variant Type, and SNV Class. Lower panel from left to right: Variants per sample and Variant classification summary.

**A**


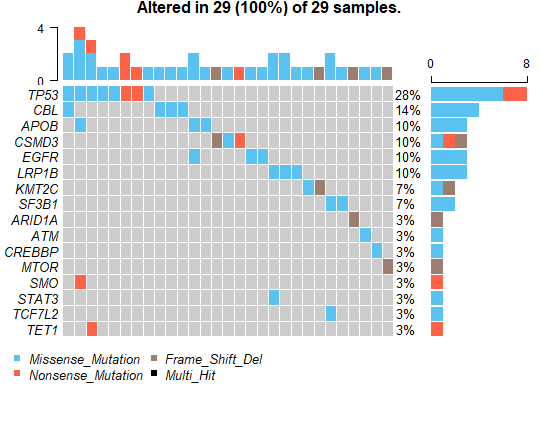


**B**


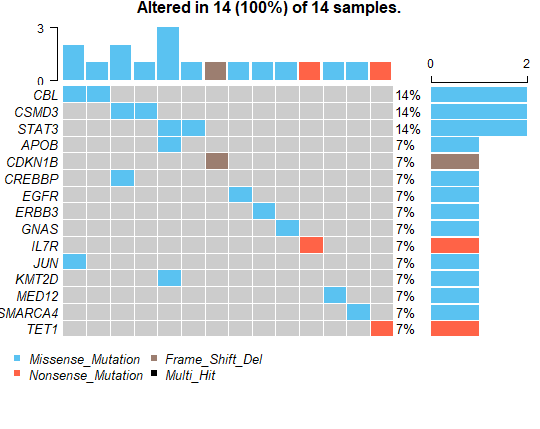


**Supplementary Figure 6: Filtered cfDNA somatic variants that were shared by WBC samples.** (A) Oncoplot of the 40 filtered mutations in 29 LC patient samples. (B) Oncoplot of the 18 filtered mutations in 14 BLN patient samples. Upper panel: mutation number per sample. Right panel: number and percentage of samples harbored mutations of this gene. Variant classification was represented by different colors as demonstrated at the lower left corner.

**A**

**
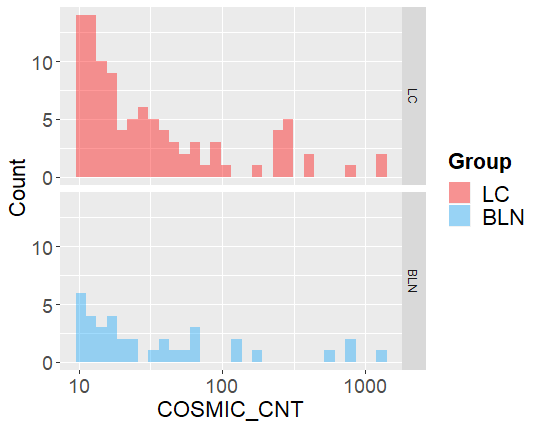
**

**B**


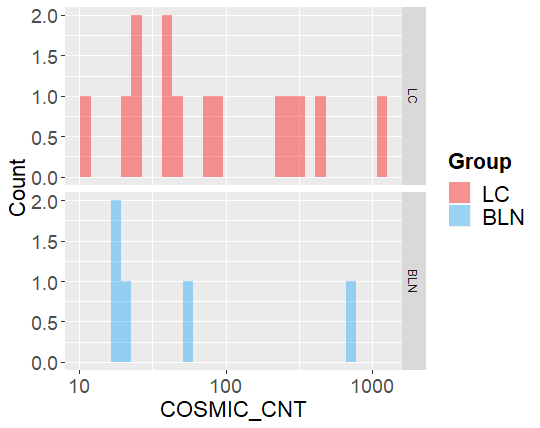


**Supplementary Figure 7: COSMIC hotspot mutations detected in WBC gDNA.** (A) Counts of all COSMIC hotspot mutations detected in LC (red) and BLN (blue) WBC gDNA. (B) Counts of COSMIC hotspot mutations detected simultaneously in matched cfDNA and WBC gDNA samples. COSMIC_CNT (x-axis, log scale): number of samples in the COSMIC database that harbor this mutation.

**Supplementary Figure 8: Median AF (y-axis) of cfDNA samples from LUSC and LUAD patients in relation to tumor volume (x-axis, log scale).** Each point with error bars represents the median and range of one cfDNA sample.

**A**

**B**

**C**

**D**

**E**

**Supplementary Figure 9: Variant distributions of the top 5 most mutated genes identified in LC plasma cfDNA.**


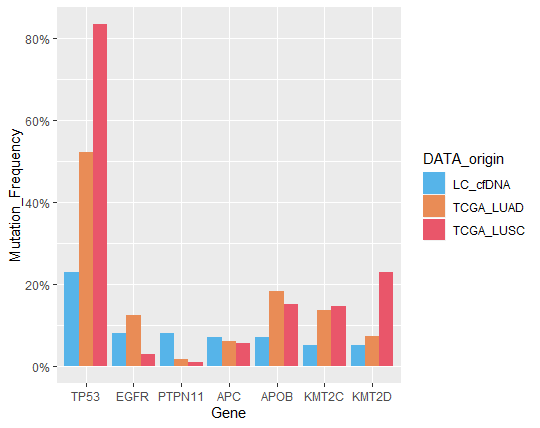


**Supplementary Figure 10:** **Mutation frequencies of the top mutated genes in LC cfDNA and corresponding frequencies in TCGA data.** TCGA data were from the PanCancer Atlas.

**A**

**
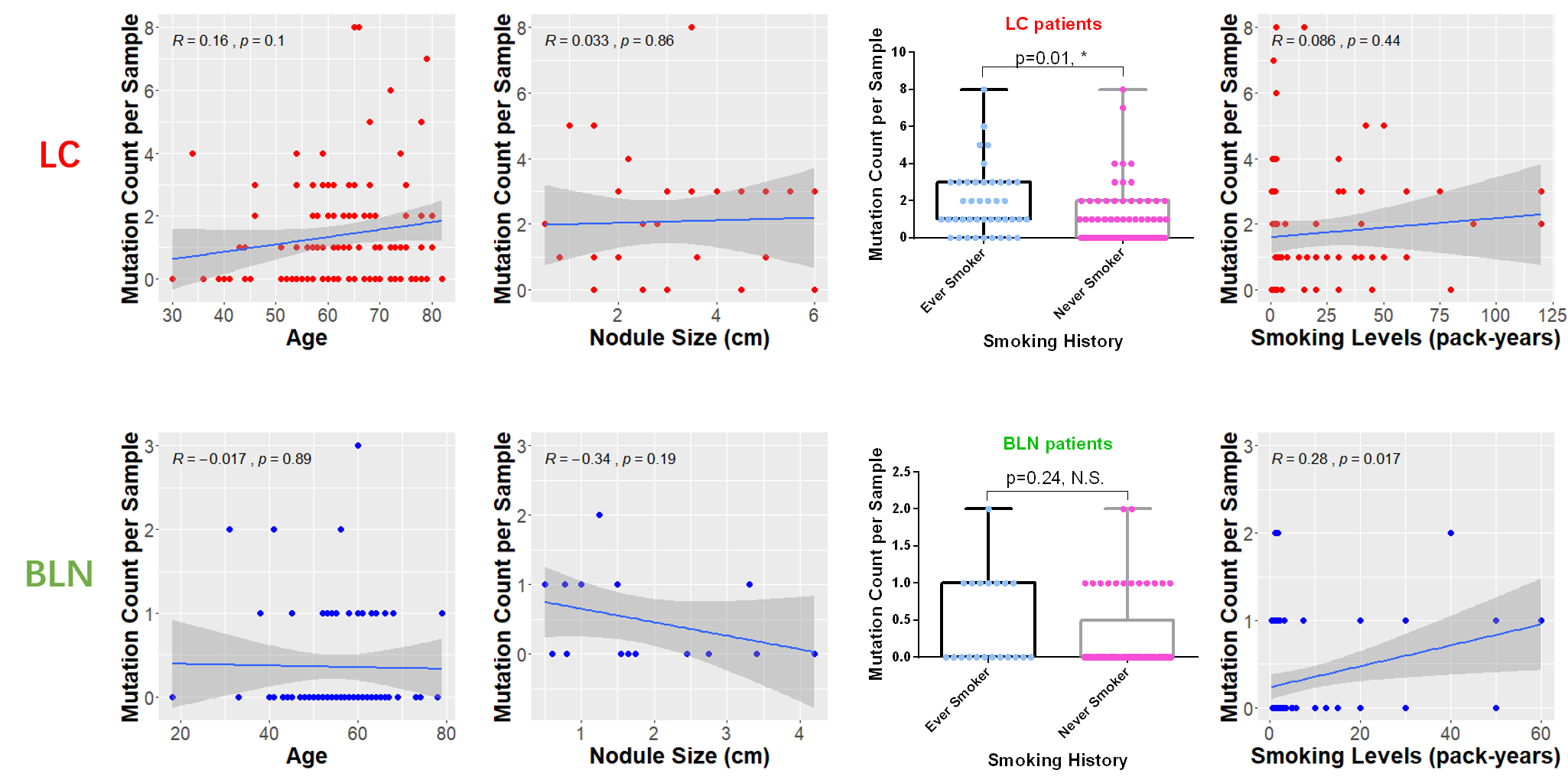
**

**B**

**
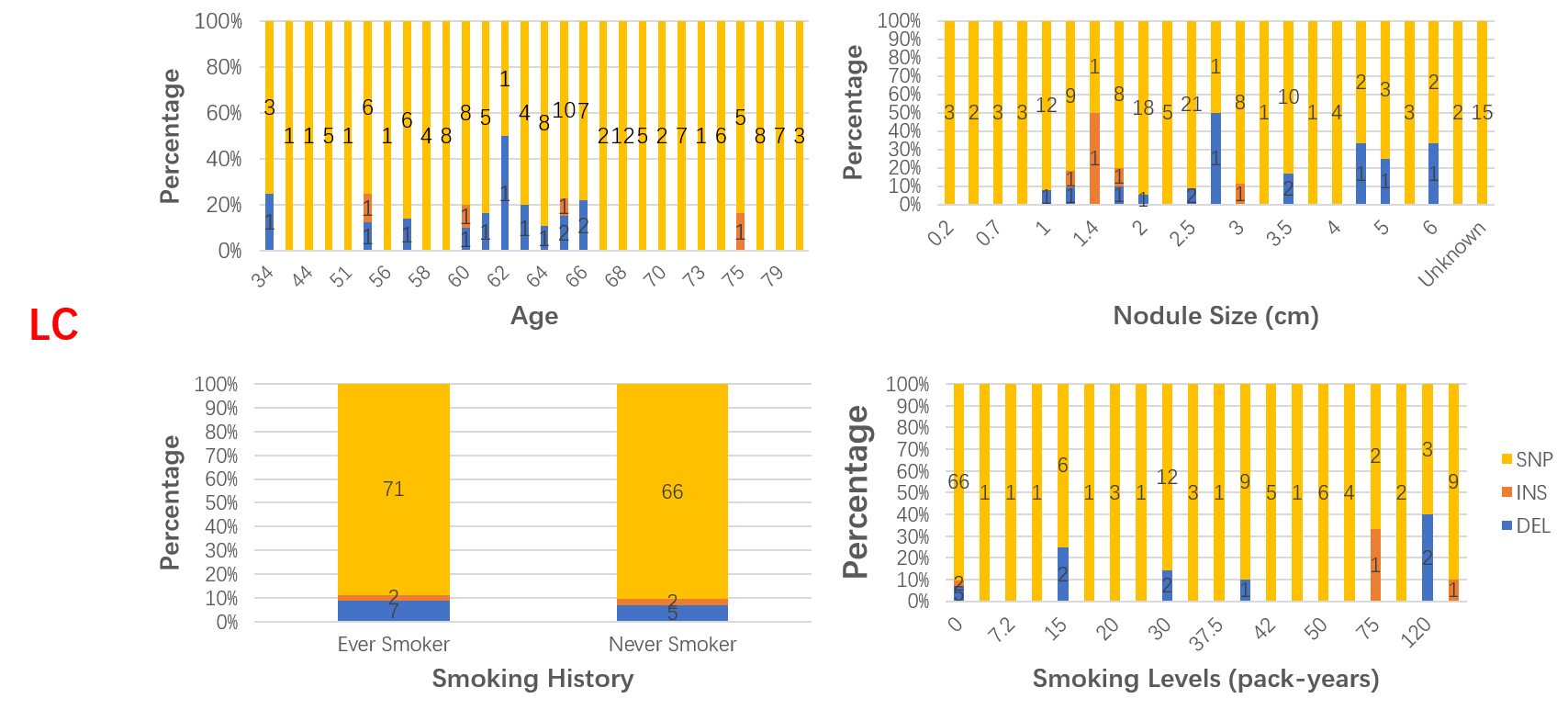
**

**
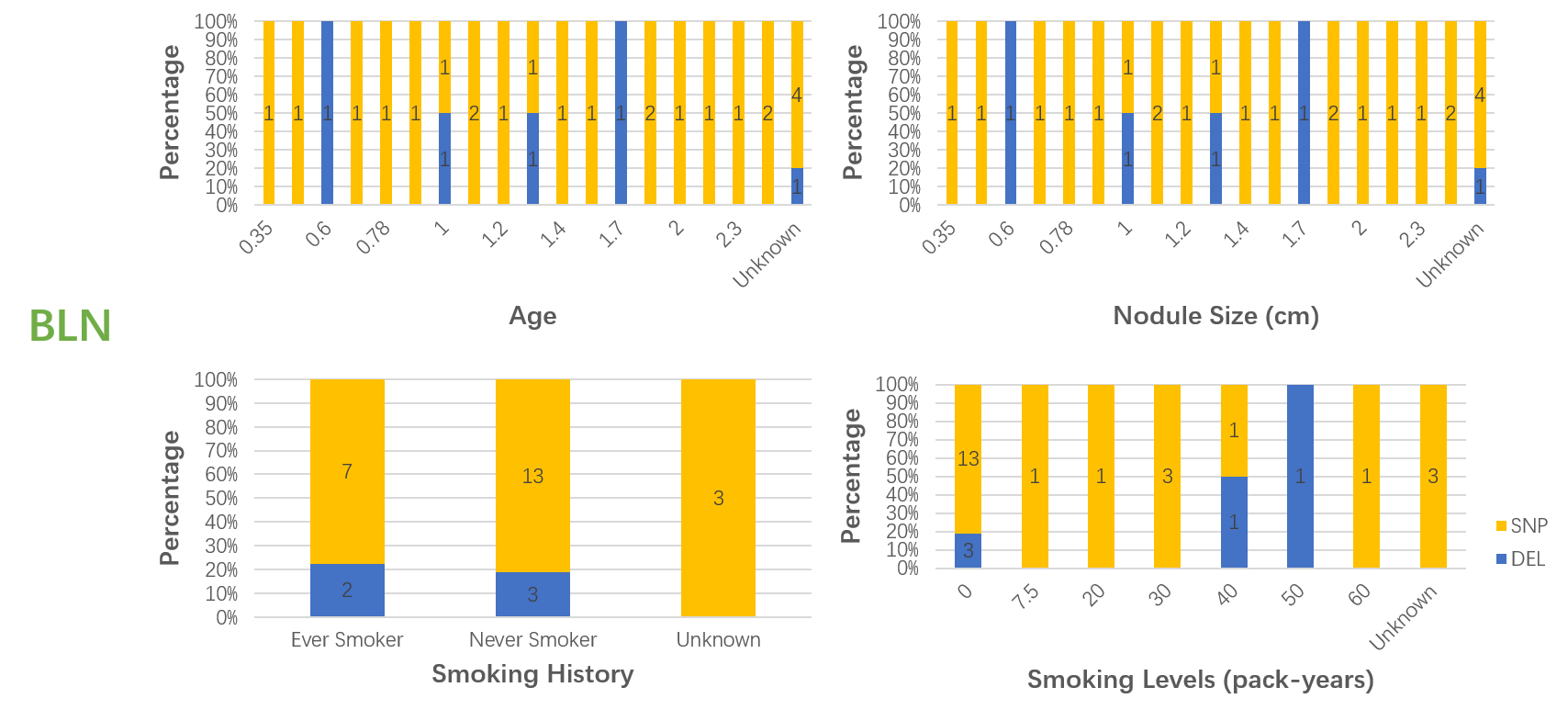
**

**Supplementary Figure 11: Impact of clinicopathological characteristics on** **(A) gene mutation burden and (B) mutation type in LC (upper panel) and BLN (lower panel) groups.**

**A**


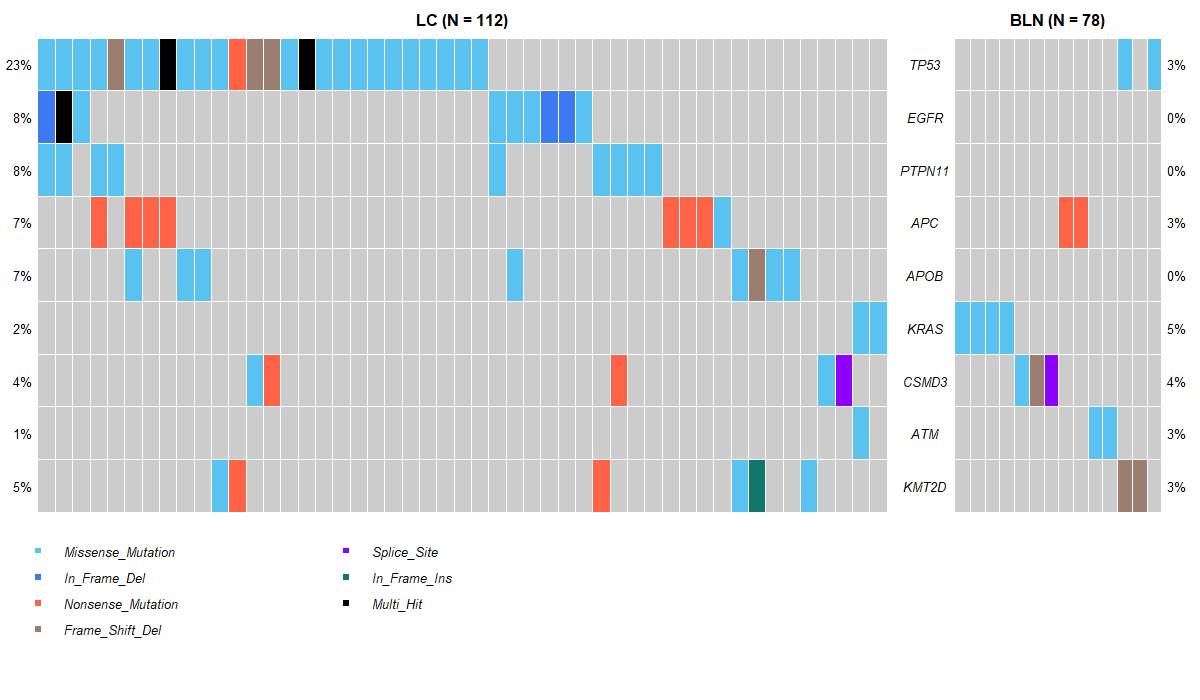


**B**

**
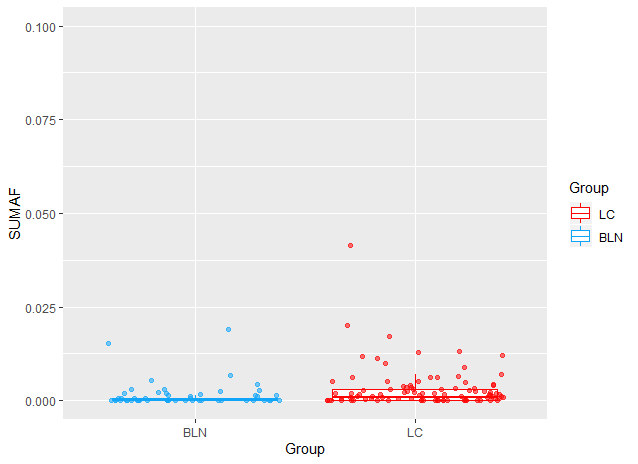
**

**C**

**
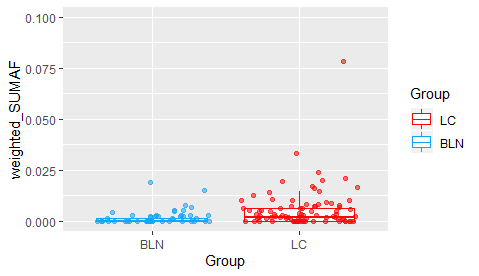
**

**Supplementary Figure 12: Comparison of plasma cfDNA mutation spectrum and mutation burden between LC and BLN patients.** (A) Co-Oncoplot of the top 5 mutated genes from LC (left) and BLN (right) patients respectively (with one gene *APC* overlap). Variant classification was represented by different colors as demonstrated at the lower left corner. (B) SUMAF and (C) weighted_SUMAF of LC and BLN cfDNA. Each point represents one cfDNA sample.


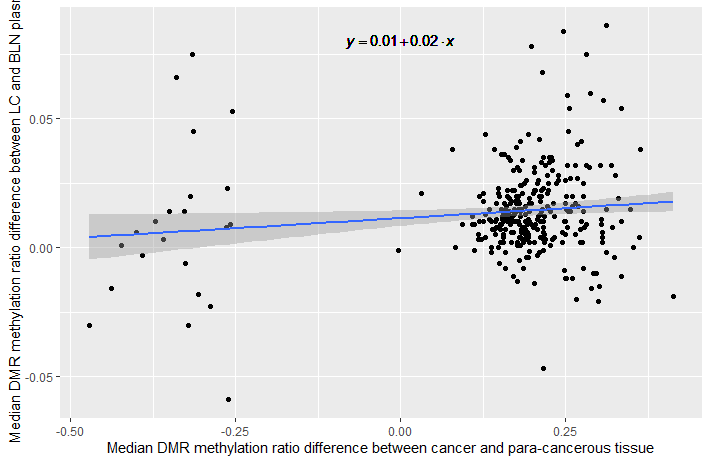


**Supplementary Figure 13: Scatter plot to evaluate the tissue-plasma concordance of identified DMRs.** X axis represents median difference of methylation levels between paired cancer and NAT. Y axis represents median methylation level difference for LC and BLN plasma.

**A**


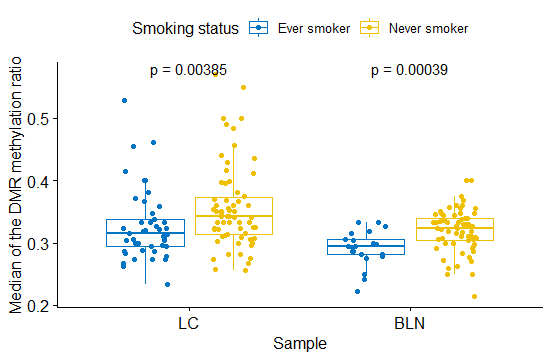


**B**

**Supplementary Figure 14: (A) Median DMR methylation ratio of plasma cfDNA and (B) CEA concentration in LC and BLN patients stratified by smoking status.**

**
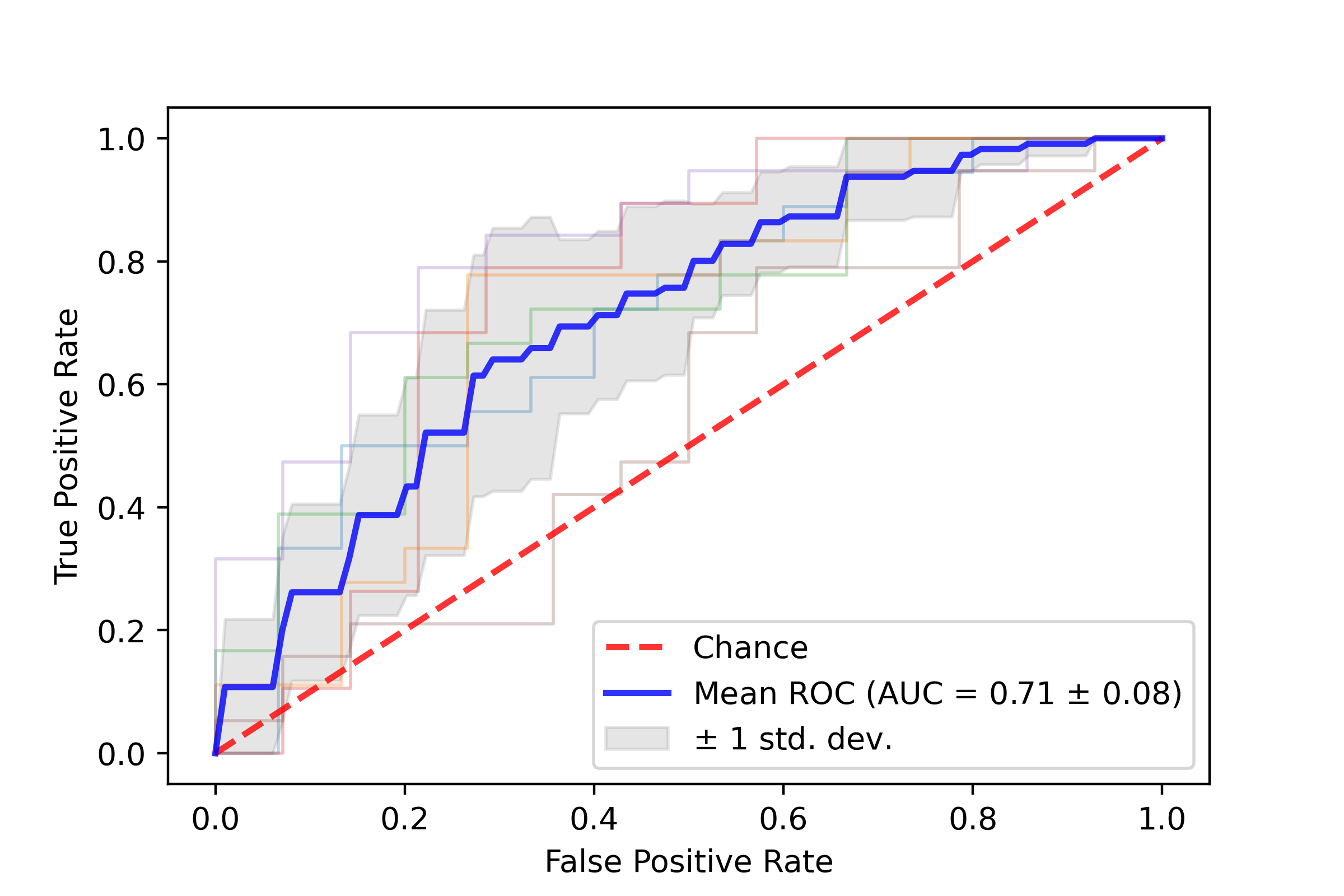
**

**Supplementary Figure 15: Predictive models based on 293 hypermethylated DMRs to distinguish LC (n=111) and BLN (n=87) plasma cfDNA.**

**
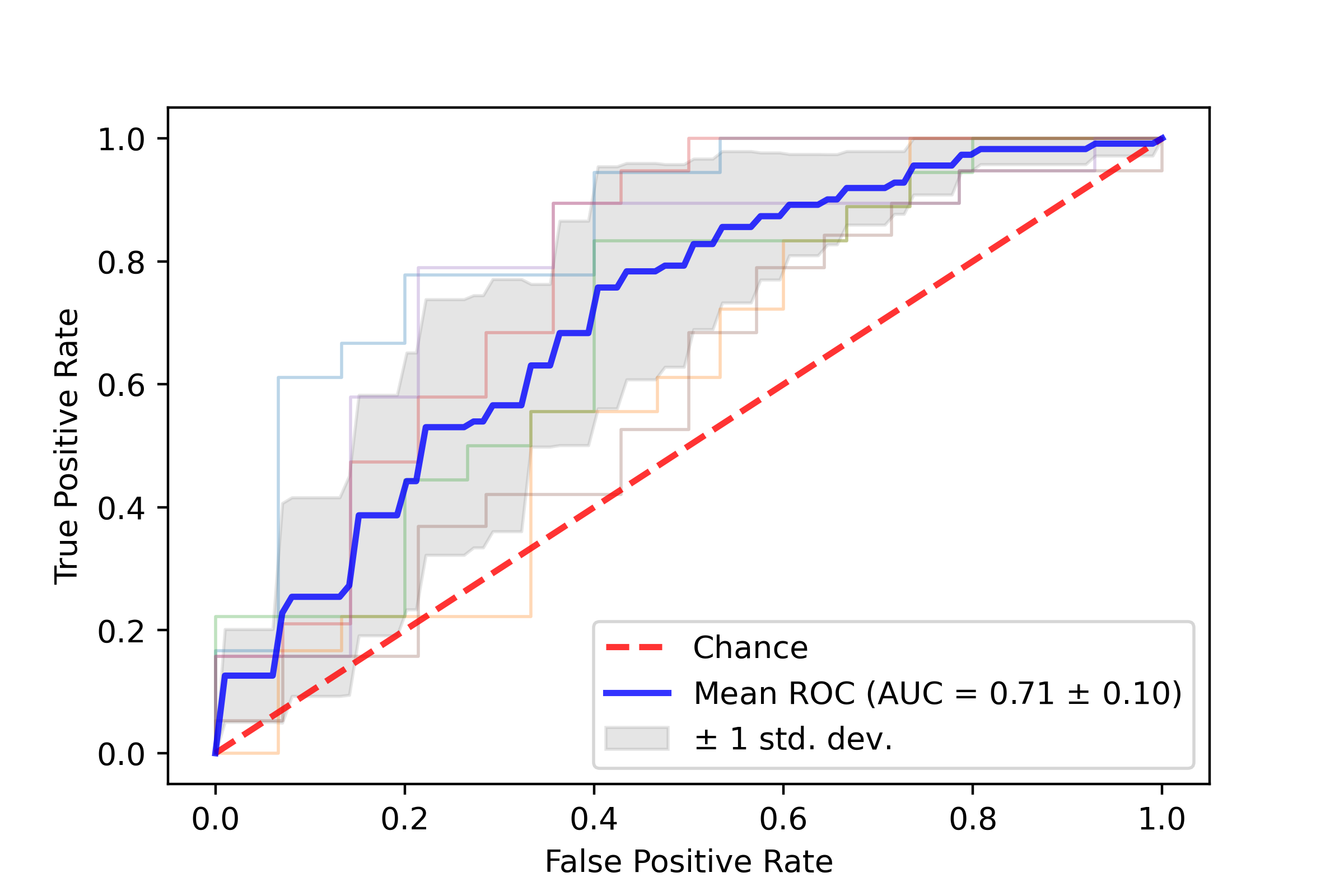
**

**Supplementary Figure 16: Predictive models to distinguish LC (n=111) from BLN (n=87) based on cfDNA methylation level with selected features (feature importance ≥ 0.01).** A total of 47 DMRs were retained in the final model. The AUC is 0.71 with 74.9% sensitivity and 58.9% specificity.

**A**

**
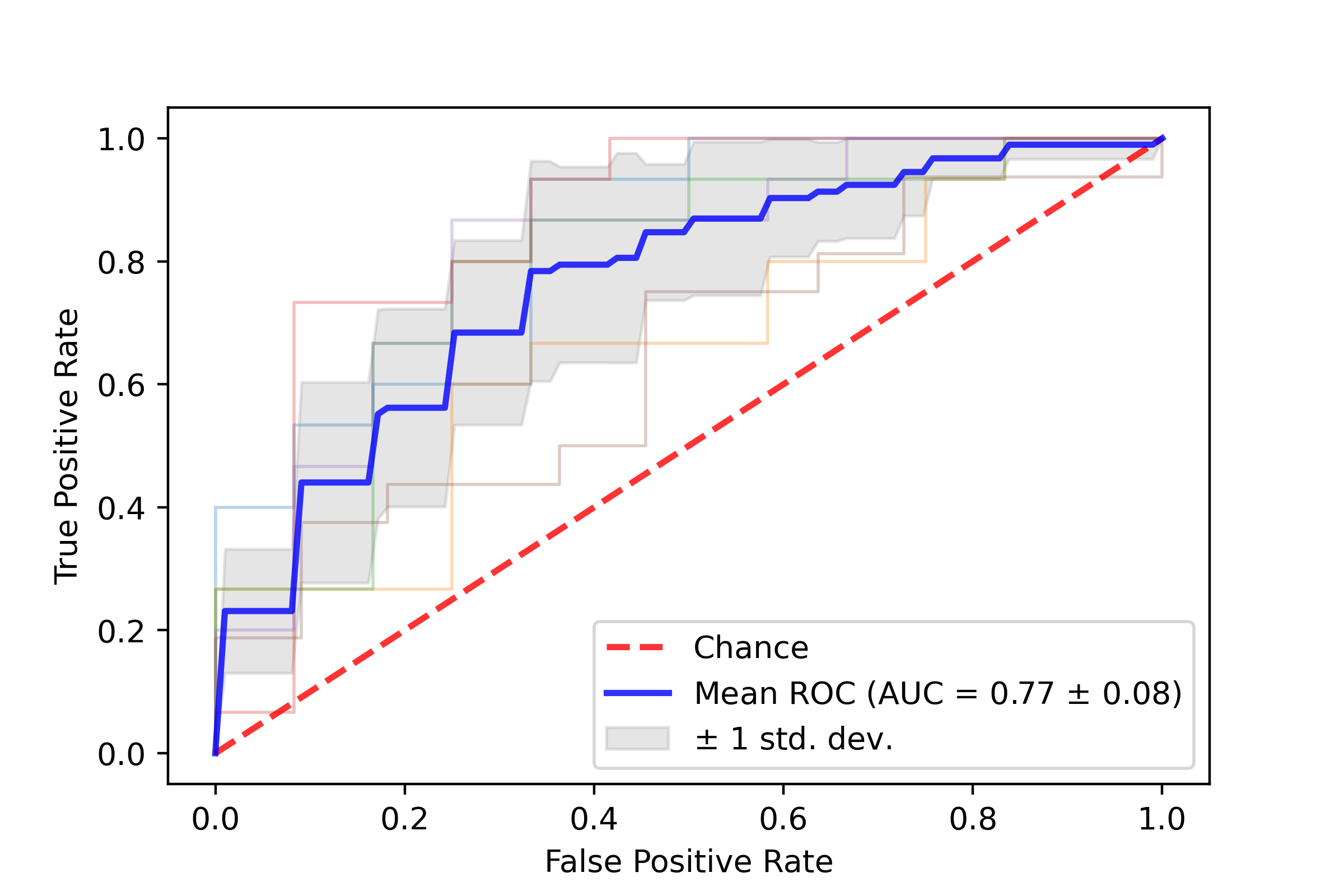
**

**B**

**
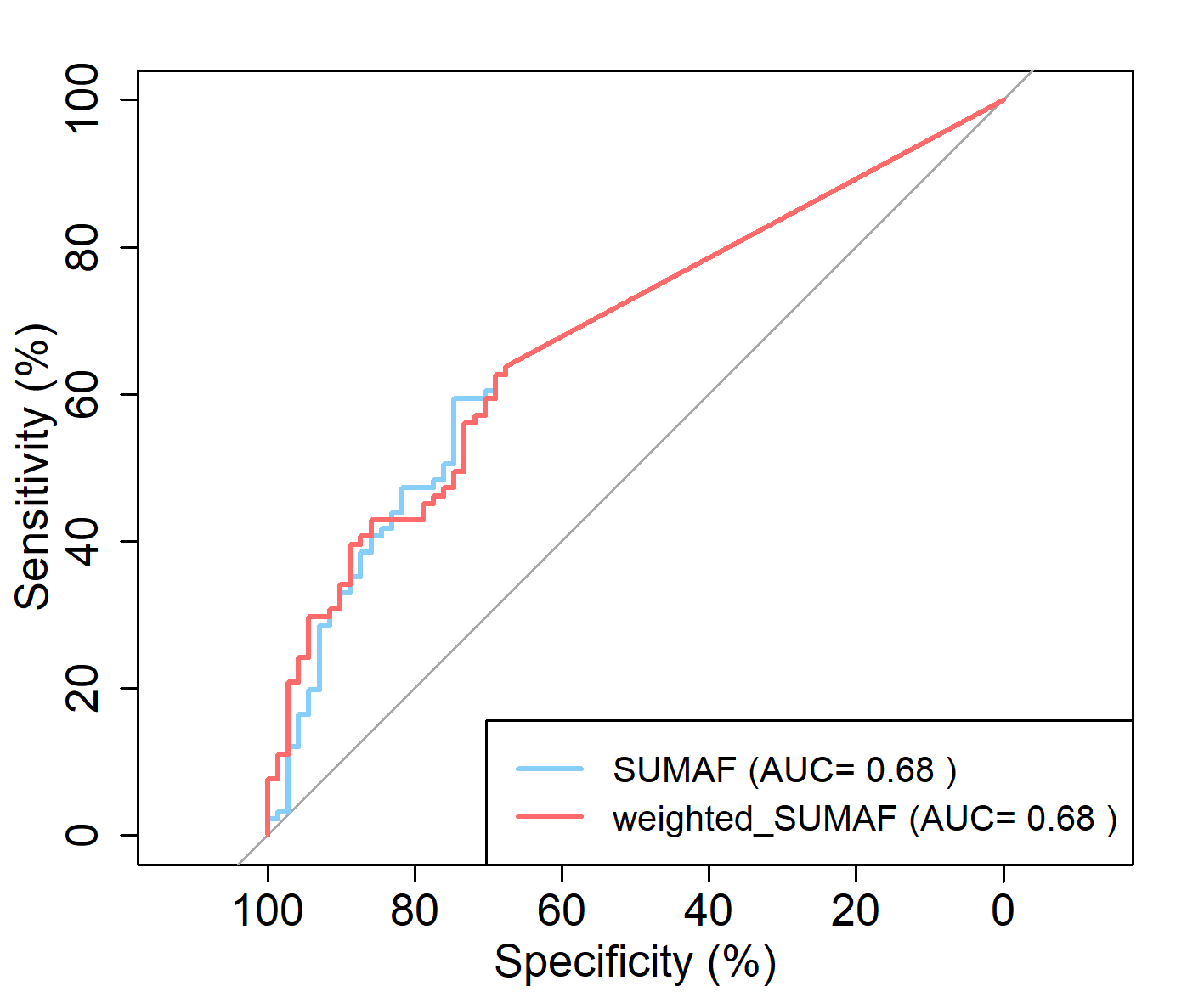
**

**C**

**
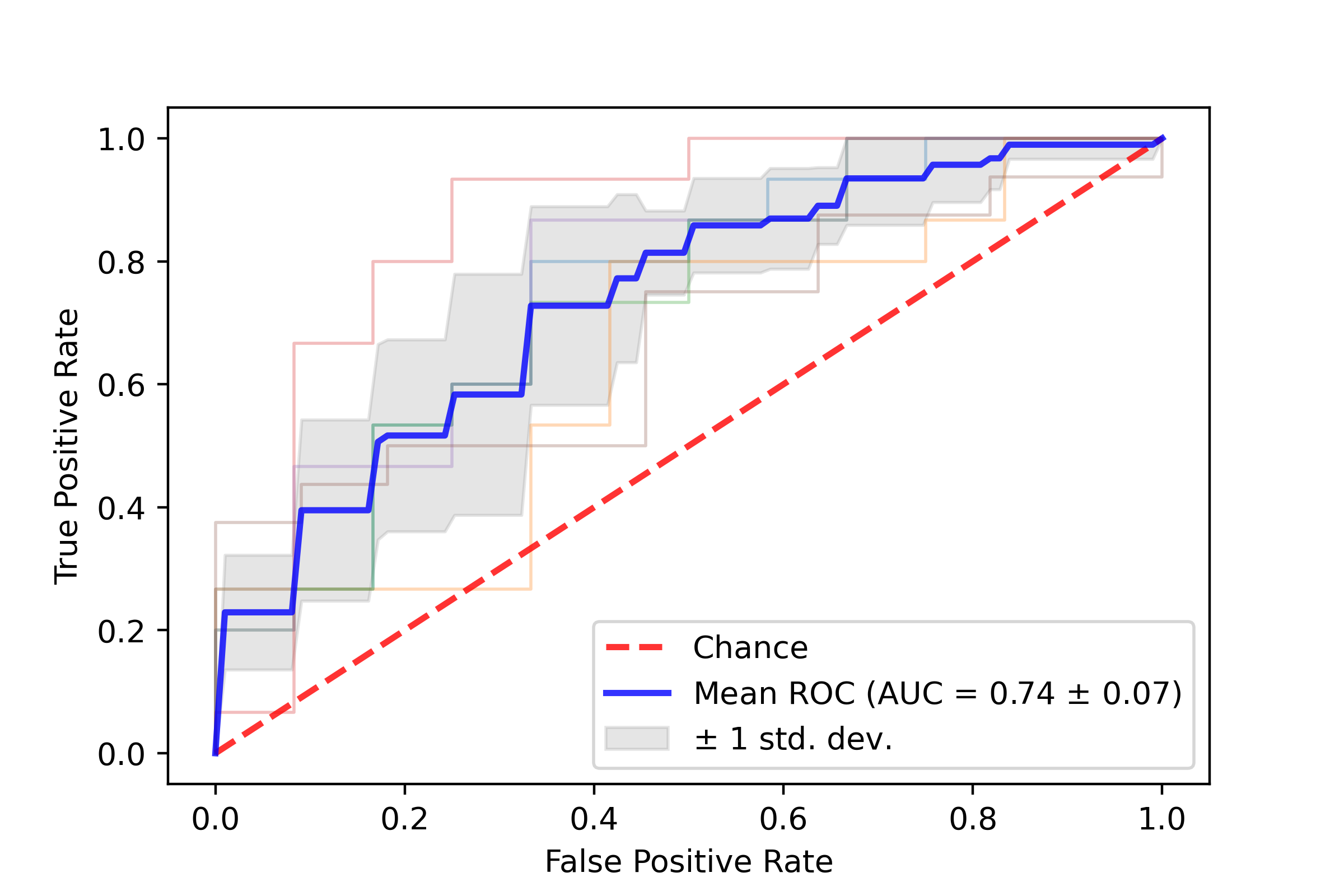
**

**Supplementary Figure 17: Bi-omics predictive models to distinguish LC (n=91) from BLN (n=71) plasma.** (A) Models built based on mutation score and selected DMR. (B) Classification models built based on mutation status alone. (C) Models built based on selected DMRs alone.

**A**

**B**

**C**

**D**

**E**

**Supplementary Figure 18: Comparison of levels of 5 serum protein markers in LC and BLN patients (**Student's t-test)**.** Dashed lines represent the threshold for each marker commonly used in clinic.


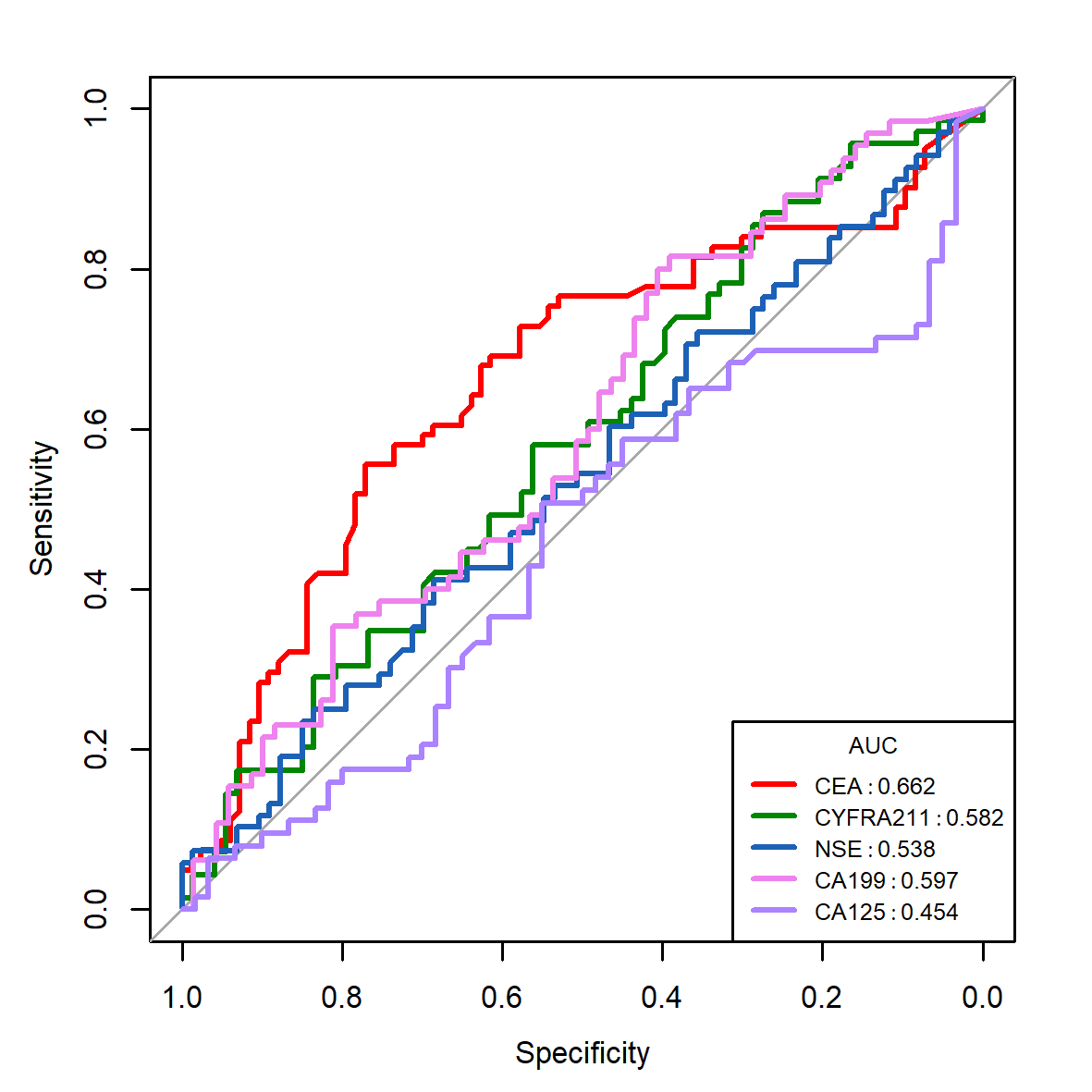


**Supplementary Figure 19: Predictive models based on single serum protein marker to distinguish LC and BLN plasma cfDNA.**

**A**


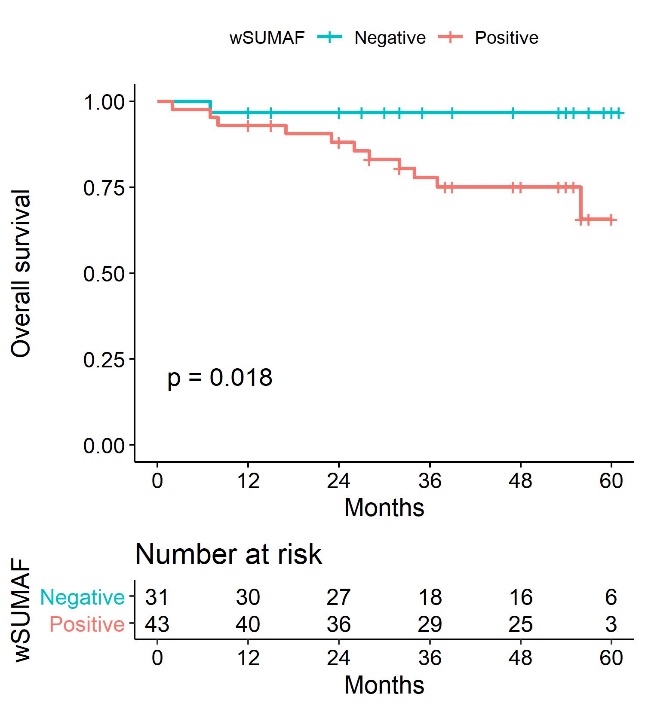


**B**


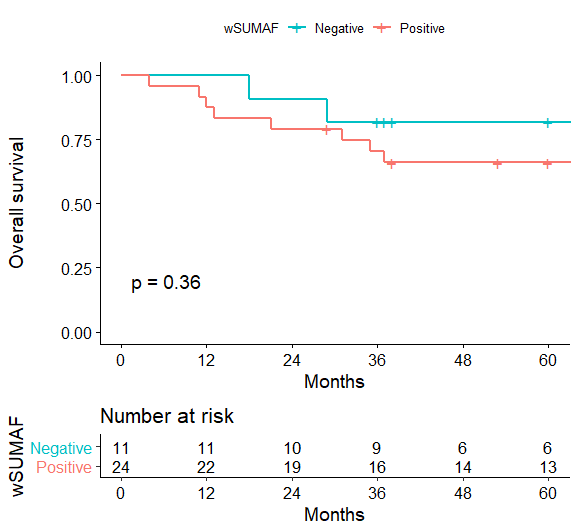


**Supplementary Figure 20: Kaplan-Meier plot on mutation-based lung cancer prognostic model in (A) stage I patients and (B) stage II-IV patients**: mutation scores in relation to OS.


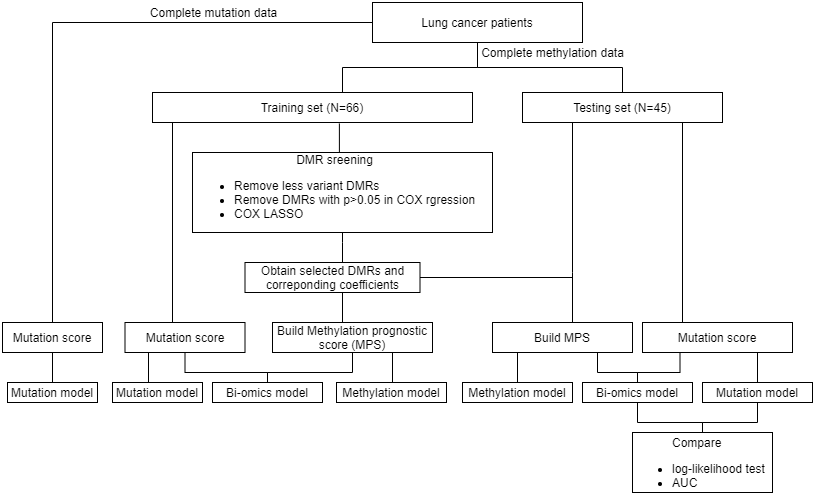


**Supplementary Figure 21: Flowchart of analysis procedure on the omics-based predictive model on lung cancer prognosis**

**A**


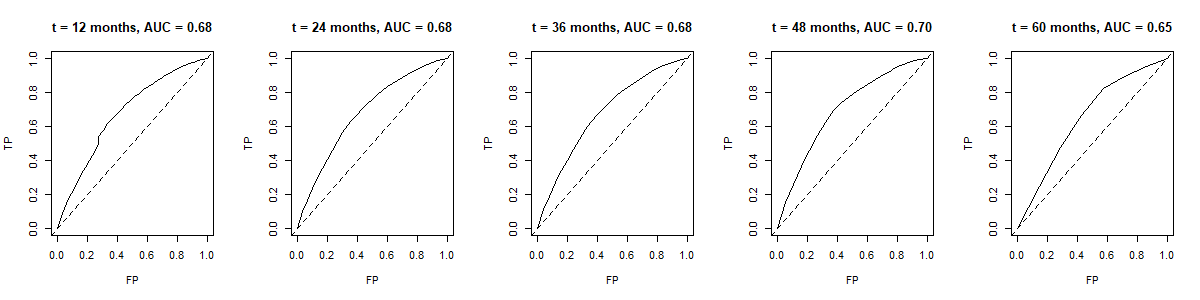


**B**


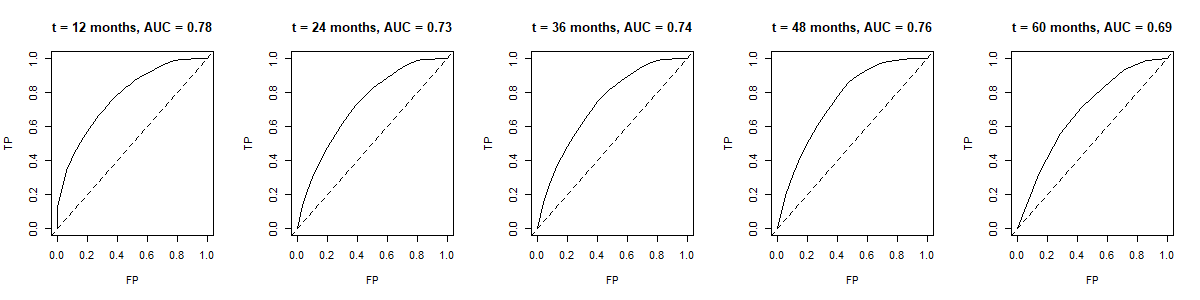


**Supplementary Figure 22: AUC of prognosis prediction models at different time point in the testing set.** (A) Mutation based model on OS; (B) Bi-omics based model on OS.
