# Supplementary material for: Non-invasive lung cancer diagnosis and prognosis based on multi-analyte liquid biopsy
Source: Mol Cancer. 2021 Jan 29;20:23. doi: 10.1186/s12943-021-01323-9 (PMC7844900; doi:10.1186/s12943-021-01323-9)
Supplement: Supplementary file 3 — Additional file 3. Methods. [file 12943_2021_1323_MOESM3_ESM.docx]

**Methods**

**Patients enrolled and samples collected in this study**

Between December 2013 and December 2018, 128 LC and 94 BLN patients were enrolled in this study at the Peking University People's Hospital, Beijing, China, with the informed consent form signed by every participant. This study was approved by the Ethics Committee of Peking University People’s Hospital (No.2017PHB106-01). The histopathological classification was based on the 2015 World Health Organization classification [1]. 4-8 mL blood was collected from the participants before surgery into 10 mL K2EDTA tubes (BD, 366643) and stored at room temperature. Plasma separation was performed within 4 hours after collection by centrifugation at 1,600×g for 10 minutes and then at 16,000×g for another 10 minutes at room temperature. Separated plasma was stored at -80 °C until DNA extraction. 25 pairs of lung cancer tissues and adjacent normal tissues were collected during surgery at stored at -80 °C.

**DNA Extraction and Quality Control**

Plasma cfDNA extraction was conducted by MagPure Circulating DNA Maxi Kit (Magen, 12917PC-100) following the manufacturer’s instructions with some modifications. The concentration of cfDNA was measured using the Qubit™ dsDNA HS Assay Kit (Thermo Fisher Scientific, Q32854). The quality of cfDNA was analyzed by Agilent High Sensitivity DNA Kit (Agilent Technologies, 5067-4626) and Agilent 2100 Bioanalyzer (Agilent Technologies). cfDNA samples with excessive high molecular weight nucleic acids were considered as contaminated by white blood cell genomic DNA (WBC gDNA) and were excluded from further analysis. gDNA was extracted from WBC, lung cancer tissues, and normal tissue adjacent to the tumor (NAT) using MagPure Buffy Coat DNA Midi KF Kit (Magen, D3537-02) per manufacturer’s instruction, and DNA concentration was measured by Qubit™ dsDNA HS Assay Kit.

**Capture panel design for targeted ultra-deep Next Generation Sequencing (NGS)**

We used a 139-gene pan-cancer panel for targeted ultra-deep sequencing. Targeted genes and exons were selected based on mutation frequency in the The Cancer Genome Atlas (TCGA) database [2] and the COSMIC database of somatic mutations in cancer [3], prioritizing cancer driver genes [4]), and exons with TCGA or COSMIC hotspot mutations.

**Library preparation for targeted ultra-deep NGS**

To reduce noises that may derive from PCR and/or sequencing errors, we used a duplex unique molecular identifier (UMI) strategy in library preparation, adapted from a previous study [5]. Briefly, cfDNA was end-repaired and ligated to sequencing adapters, and index PCR was performed followed by purification by Agencourt AMPure XP beads (Beckman Coulter, A63882). WBC gDNA was processed in the same way except for it was fragmented by sonication before library preparation.

Target capture reactions were performed using xGen® Lockdown® Reagents (IDT technologies) per manufacturer’s instruction. Captured Libraries were amplified in a 50 μL PCR mix composed of 25 μL 2× KAPA HiFi Hot Start Ready Mix, 5 μL PCR primer pair (10 μM) and 20 μL beads suspensions with the following cycling conditions: 45s at 98°C, followed by 13 cycles of 98°C for 15 s, 60°C for 30 s, and 72°C for 30 s; final extension was performed at 72°C for 1min. Libraries were purified by Agencourt AMPure XP beads, quantified by Qubit™ dsDNA HS Assay Kit, and sequenced on MGISEQ-2000 (MGI Tech) using 2×100 paired-end sequencing.

**Library preparation for targeted bisulfite sequencing**

To improve the quality of cfDNA whole-genome bisulfite sequencing (WGBS) libraries, we adopted a single-stranded DNA (ssDNA) library preparation strategy. Briefly, bisulfite conversion was performed on input DNA using EZ DNA Methylation-Gold™ Kit (Zymo Research, D5006) per manufacturer's instructions. Next, bisulfite-converted ssDNA was ligated to sequencing adaptors as described previously [6]. gDNA extracted from lung cancer or normal tissues was fragmented by sonication before library preparation.

Targeted capture reactions of the WGBS libraries were performed using SeqCap Epi CpGiant Probes (Roche) following the manufacturer's instruction. Captured libraries were amplified and sequenced on MGISEQ-2000 using 2×100 paired-end sequencing.

**Variant analysis**

Targeted sequencing data from cfDNA libraries were processed as follows: UMI sequences were trimmed from fastq data using in-house scripts and were adapter trimmed and quality trimmed using SOAPnuke-2.0.3 [7]. Reads were aligned against the human reference genome (hg19) using BWA-MEM (version 0.7.17) [8]. Candidate mutations were identified from the aligned reads using a two-step procedure: Firstly, hotspot mutations (defined as point mutations, small insertions and deletions represented in COSMIC database (<https://cancer.sanger.ac.uk/cosmic>, version 85) with >= 20 cancer cases) were identified using the in-house script and filtered using an allele fraction cutoff of 0.05% (except for indels, which were not filtered). Secondly, non-hotspot mutations were identified using freebayes (version 1.1.0) [9] and filtered using an allele fraction cutoff of 0.05%. These two sets of variants were combined and filtered for potential germline variants (with allele fraction >=25%) [10]. Variants were further filtered for germline mutations using a custom germline database derived from the ExAC germline variants data [11] and 1000 Genome data [12], as well as a custom false-positive database. Remaining variants were then annotated using VEP (version 95.2-0) [13]. For cfDNA samples, variants were further filtered using the following set of criteria: variants were first filtered to exclude intronic and silent mutations. For the remaining hotspot variants, only those with at least 3 supporting UMI families and at least one supporting duplex UMI family were retained (except for indels). For the remaining non-hotspot variants, only ones with at least 8 supporting UMI families and at least one supporting duplex UMI family, or ones with at least 6 supporting UMI families and at least two duplex UMI families, were retained. Non-hotspot mutations with a SIFT prediction of "tolerated" and a PolyPhen prediction of "benign" were excluded. Finally, within the remaining non-hotspot variants, only those with a SIFT score <= 0.02 and a PolyPhen score >= 0.95, or a PolyPhen score of 1, or a SIFT score of 0, were retained. For WBC samples, no further filtering was applied. To derive the final set of variants for plasma sample, cfDNA variants were filtered with variants identified from the matched WBC sample.

**Mutation scoring system**

Variants were classified and weighted according to the following arbitrarily defined tiered scoring system: COSMIC hotspots with more than 500 cancer cases were given a score of 8; TCGA hotspot variants [14] or COSMIC hotspots with more than 100 cancer cases and not in the former class were given a score of 4; COSMIC hotspots with more than 20 cancer cases and not in the former class were given a score of 2; the rest of variants were given a score of 1.

**Methylation data analysis**

Targeted bisulfite sequencing data were processed as follows. First, low-quality reads and 3’ sequencing adapters were trimmed by fastp (version 0.19.7) [15]. Then, pair-end reads were aligned to the hg19 reference genome using BitMapperBS (version 1.0.0.8) [16]. Only reads mapped in proper pair to a unique genomic position and spanning an insert size between 30 bp and 500 bp were retained. Next, duplicates were marked with sambamba (v0.6.8) [17]. Finally, methylation rates were calculated as #C/(#C+#T) for individual CpG sites with at least 4x coverage using MethylDackel (<https://github.com/dpryan79/MethylDackel>, version 0.3.0).

**Identification of differentially methylated regions (DMRs)**

A Bayesian hierarchical model was used to detect the differential methylated loci between 25 lung cancer tissues and 25 matched normal tissues (p<0.001 and delta>0.2) [18]. To account for the spatial correlation of methylation ratio, smoothing was applied to combine the information from proximal CpG sites to identify differentially methylated regions (DMRs). DMRs were defined as the regions satisfying the following criteria: ≥50bp, containing ≥3 CpG sites within the region, and ≥80% CpG sites with significant p-values. Only hypermethylated DMRs were used in the subsequent analysis.

**Predictive model construction**

Regional methylation ratio was calculated per DMR for each cfDNA sample sequenced by targeted bisulfite sequencing and processed as features by dividing the sum of methylated cytosine by the sum of depth in the DMR. Six-fold cross-validation was performed to validate random forest models for classifying plasma cfDNA of lung cancer patients from that of patients bearing benign lung nodules using the python package scikit-learn [19].

Feature selections were performed on the training data only, using a feature importance cutoff of 0.01. Random forest models were fitted using the selected DMRs with the parameters: number of trees=60, depth=5. The fitted models were then applied in the validation set from which the sensitivity, specificity, and area under the curve (AUC) were calculated. Multi-omics prediction models were trained and validated similarly, except that feature selections were applied to the DMR features only.

**Identification and validation of prognostic markers**

To identify methylation-based prognostic markers, samples were randomly divided into a training set and testing set using a 60/40 split. We applied the following procedure to select the potential methylation-related prognostic factors and to fit prognosis model in the training set: we first removed DMRs with a standard deviation<0.03 from the identified lung cancer DMRs as mentioned above since less variant features provided limited information; we then used the selected DMRs to fit a LASSO Cox proportional hazard model on OS. Through 10-fold cross-validation, we chose the tuning parameter λ when the partial likelihood deviance reached the lowest, from which DMRs were further filtered and the coefficients of the each DMR were obtained. We calculated the methylation-based prognostic score (MPS) for each individual as the sum of the products of the DMR methylation level and its coefficient and combined the mutation score (wSUMAF) with the MPS as the multi-omics score. We then assessed the association of lung cancer prognosis with mutation score and multi-omics score separately in the training set and testing set. Kaplan–Meier curves were plotted for each analysis. Finally, two separate multivariate Cox proportional hazard models were built on wSUMAF only and both wSUMAF and MPS with adjustment of age, stage, histological type and smoking status in the testing set. To avoid information loss through categorization, both wSUMAF and MPS were analyzed as continuous variables in the multivariate Cox regression. To compare the performance of two models, the incident cases / dynamic controls ROC curve was plotted [20]. The R package of glmnet, survival, survminer (https://CRAN.R-project.org/package=survminer), risksetROC were used. The analyses procedure is summarized in the Supplementary Figure 18.

**References:**

1. Travis, W. D., Brambilla, E., Burke, A. P., Marx, A., & Nicholson, A. G. (2015). Introduction to The 2015 World Health Organization Classification of Tumors of the Lung, Pleura, Thymus, and Heart. *Journal of Thoracic Oncology, 10*(9), 1240-1242.

2. Kandoth, C., McLellan, M. D., Vandin, F., Ye, K., Niu, B., Lu, C., . . . Ding, L. (2013). Mutational landscape and significance across 12 major cancer types. *Nature, 502*(7471), 333-339.

3. Tate, J. G., Bamford, S., Jubb, H. C., Sondka, Z., Beare, D. M., Bindal, N., . . . Forbes, S. A. (2018). COSMIC: the Catalogue Of Somatic Mutations In Cancer. *Nucleic Acids Research, 47*(D1), D941-D947.

4. Bailey, M. H., Tokheim, C., Porta-Pardo, E., Sengupta, S., Bertrand, D., Weerasinghe, A., . . . Ding, L. (2018). Comprehensive Characterization of Cancer Driver Genes and Mutations. *Cell, 173*(2), 371-385.e318.

5. Newman, A. M., Lovejoy, A. F., Klass, D. M., Kurtz, D. M., Chabon, J. J., Scherer, F., . . . Alizadeh, A. A. (2016). Integrated digital error suppression for improved detection of circulating tumor DNA. *Nat Biotechnol, 34*(5), 547-555.

6. Gansauge, M.-T., Gerber, T., Glocke, I., Korlevic, P., Lippik, L., Nagel, S., . . . Meyer, M. (2017). Single-stranded DNA library preparation from highly degraded DNA using T4 DNA ligase. *Nucleic acids research, 45*(10), e79-e79.

7. Chen, Y., Chen, Y., Shi, C., Huang, Z., Zhang, Y., Li, S., . . . Chen, Q. (2017). SOAPnuke: a MapReduce acceleration-supported software for integrated quality control and preprocessing of high-throughput sequencing data. *GigaScience, 7*(1).

8. Li, H., & Durbin, R. (2009). Fast and accurate short read alignment with Burrows–Wheeler transform. *Bioinformatics, 25*(14), 1754-1760.

9. Garrison, E., & Marth, G. (2012). Haplotype-based variant detection from short-read sequencing. *arXiv, 1207*.

10. Phallen, J., Sausen, M., Adleff, V., Leal, A., Hruban, C., White, J., . . . Velculescu, V. E. (2017). Direct detection of early-stage cancers using circulating tumor DNA. *Science Translational Medicine, 9*(403), eaan2415.

11. McVean, G. A., Altshuler, D. M., Durbin, R. M., Abecasis, G. R., Bentley, D. R., Chakravarti, A., . . . University of, G. (2012). An integrated map of genetic variation from 1,092 human genomes. *Nature, 491*(7422), 56-65.

12. Siva, N. (2008). 1000 Genomes project. *Nature Biotechnology, 26*(3), 256-256.

13. McLaren, W., Gil, L., Hunt, S. E., Riat, H. S., Ritchie, G. R. S., Thormann, A., . . . Cunningham, F. (2016). The Ensembl Variant Effect Predictor. *Genome Biology, 17*(1), 122.

14. Bailey, M. H., Tokheim, C., Porta-Pardo, E., Sengupta, S., Bertrand, D., Weerasinghe, A., . . . Ding, L. (2018). Comprehensive Characterization of Cancer Driver Genes and Mutations. *Cell, 173*(2), 371-385.e318.

15. Chen, S., Zhou, Y., Chen, Y., & Gu, J. (2018). fastp: an ultra-fast all-in-one FASTQ preprocessor. *Bioinformatics, 34*(17), i884-i890.

16. Cheng, H., & Xu, Y. (2018). BitMapperBS: a fast and accurate read aligner for whole-genome bisulfite sequencing. *bioRxiv*, 442798.

17. Tarasov, A., Vilella, A. J., Cuppen, E., Nijman, I. J., & Prins, P. (2015). Sambamba: fast processing of NGS alignment formats. *Bioinformatics, 31*(12), 2032-2034.

18. Wu, H., Xu, T., Feng, H., Chen, L., Li, B., Yao, B., . . . Conneely, K. N. (2015). Detection of differentially methylated regions from whole-genome bisulfite sequencing data without replicates. *Nucleic Acids Research, 43*(21), e141-e141.

19. Pedregosa, F., Varoquaux, G., Gramfort, A., Michel, V., Thirion, B., Grisel, O., . . . Dubourg, V. J. J. o. m. l. r. (2011). Scikit-learn: Machine learning in Python. *12*(Oct), 2825-2830.

20. Heagerty, P. J., & Zheng, Y. J. B. (2005). Survival model predictive accuracy and ROC curves. *61*(1), 92-105.
